# Oleanane triterpenoids with C-14 carboxyl group from *Astilbe grandis* inhibited LPS-induced macrophages activation by suppressing the NF-*κ*B signaling pathway

**DOI:** 10.3389/fphar.2024.1413876

**Published:** 2024-08-01

**Authors:** Lan Yue, Jinfang Luo, Chenliang Zhao, Jinfeng Zhao, Jianghai Ye, Kang He, Juan Zou

**Affiliations:** ^1^ School of Pharmacy, Guizhou University of Traditional Chinese Medicine, Guiyang, China; ^2^ School of Basic Medicine, Guizhou University of Traditional Chinese Medicine, Guiyang, China

**Keywords:** *Astilbe grandis*, oleanane triterpenoids, 3α-acetoxyolean-12-en-27-oic acid, 3β-acetoxyolean-12-en-27-oic acid, anti-inflammatory, RAW 264.7 cells, THP-1 cells, NF-κB

## Abstract

**Background:**

Excessive inflammation poses significant risks to human physical and mental health. *Astilbe grandis*, a traditional Miao medicine, is renowned for its anti-inflammatory properties. However, the specific anti-inflammatory effects and mechanisms of many compounds within this plant remain unclear. This study aims to investigate the anti-inflammatory effects and mechanisms of two characteristic oleanane triterpenoids, 3*α*-acetoxyolean-12-en-27-oic acid (**1**) and 3*β*-acetoxyolean-12-en-27-oic acid (**2**), isolated from *Astilbe grandis*, using lipopolysaccharide (LPS)-induced Macrophages.

**Methods:**

The anti-inflammatory effects and mechanisms of compounds **1** and **2** were investigated by establishing an LPS-induced inflammation model in RAW 264.7 cells and THP-1 cells. Nitric oxide (NO) levels were assessed using the Griess method. The concentrations of tumor necrosis factor-alpha (TNF-*α*), interleukin-6 (IL-6), and interleukin-1beta (IL-1*β*) were measured via enzyme-linked immunosorbent assay (ELISA). The expression of cyclooxygenase-2 (COX-2) and inducible nitric oxide synthase (iNOS) was determined using western blotting and quantitative real-time PCR (qRT-PCR). Additionally, the phosphorylation level of p65 in nuclear factor-kappa B (NF-*κ*B) was assessed through western blotting. The nuclear translocation of NF-*κ*B p65 was assessed through immunofluorescence staining. Finally, the binding affinity of the compounds to NF-*κ*B p65 target was validated through molecular docking.

**Results:**

Compounds **1** and **2** significantly inhibited the expression of NO, TNF-*α*, IL-6, IL-1*β*, COX-2, and iNOS in LPS-induced Macrophages. Mechanistically, they attenuated the activation of the NF-*κ*B signaling pathway by downregulating the phosphorylation level and nuclear translocation of p65.

**Conclusion:**

This study elucidates the anti-inflammatory activities and potential mechanism of the characteristic oleanane triterpenoids with C-14 carboxyl group, compounds **1** and **2**, in LPS-induced Macrophages by inhibiting the NF-*κ*B signaling pathway for the first time. These findings suggest that these two compounds hold promise as potential candidates for anti-inflammatory interventions in the future.

## 1 Introduction

Inflammation is a common and complex response closely associated with many diseases. While an appropriate inflammatory response is vital for eliminating harmful stimuli and aiding tissue repair (Nunes et al., 2021), an excessive inflammatory reaction can lead to various inflammatory disorders, such as arthritis (Peeters et al., 2023), Alzheimer’s disease (Miron et al., 2018), atherosclerosis (Wang et al., 2023), and even cancer (Lin et al., 2019).

Numerous inflammatory mediators, including nitric oxide (NO), inducible nitric oxide synthase (iNOS), cyclooxygenase-2 (COX-2), tumor necrosis factor-alpha (TNF-*α*), interleukin-6 (IL-6), and interleukin-1beta (IL-1*β*), are known to increase during lipopolysaccharide (LPS)-induced inflammation (Lee et al., 2017). Particularly noteworthy is iNOS, responsible for NO production, which can elevate NO levels, leading to DNA damage, mutagenesis, and cancer progression (Ohshima and Bartsch, 1994). Macrophages, pivotal inflammatory and immune effector cells, undergo activation when exposed to external stimuli such as LPS. This activation involves the phosphorylation of NF-*κ*B p65 and the exposure of its nuclear localization signal (Zucoloto et al., 2017). Subsequently, NF-*κ*B p65 translocates into the cell nucleus, exerting a transcriptional regulatory effect that triggers the gene transcription of TNF-*α*, IL-6, IL-1*β*, iNOS, and COX-2, thereby promoting the inflammatory response (Afonina et al., 2017; Ma et al., 2022). Consequently, reducing the activation of the NF-*κ*B signaling pathway in macrophages is considered critical for preventing the onset and progression of inflammation-related diseases.

In recent years, there has been increasing attention on active compounds derived from botanical sources and traditional medicines (Zhang et al., 2019). *Astilbe grandis* Stapf ex Wils., a member of the *Astilbe* genus in the Saxifragaceae family, is a widely used traditional folk medicine known for its remarkable anti-inflammatory and analgesic effects (Jiang et al., 2006; Wang et al., 2013; He et al., 2021). Studies indicate that plants of the *Astilbe* genus primarily contain triterpenoids, coumarins, and flavonoids (He et al., 2020). Among them, bergenin is the main component of coumarins, and astilbin is the main component of flavonoids, both of which exhibit good anti-inflammatory activity (Wang et al., 2018; Yang et al., 2020). While the anti-inflammatory activity and mechanisms of coumarins and flavonoids have been extensively reported, the anti-inflammatory activity and mechanisms of triterpenoids are often overlooked. In our previous studies, various compounds were isolated from *A. grandis*, and oleanane triterpenoids with C-14 carboxyl group were identified as characteristic components of the *Astilbe* genus (He et al., 2020; He et al., 2021). Pharmacological studies revealed that these triterpenoids exhibit significant anti-tumor activity (Sun et al., 2004; Tu et al., 2006; Zhang et al., 2008), while their anti-inflammatory activity is seldom reported. Only one study demonstrated that 3*β*, 6*β*-dihydroxyolean-12-en-27-oic acid from *Astilbe chinensis* could inhibit the activity of 5-LOX, thereby showing some anti-inflammatory activity (Moon et al., 2005); however, the mechanism of this anti-inflammatory effect remains unclear.

To further explore the anti-inflammatory activity and mechanism of the triterpenoid constituents of *A. grandis*, we conducted the first investigation into the anti-inflammatory activities and mechanisms of the characteristic oleanane triterpenoids with C-14 carboxyl group, namely, 3*α*-acetoxyolean-12-en-27-oic acid (**1**) and 3*β*-acetoxyolean-12-en-27-oic acid (**2**), isolated from *A. grandis*. Our findings revealed that both compounds reduced the levels of inflammatory factors and proteins in LPS-induced RAW 264.7 cells and THP-1 cells. Furthermore, we elucidated the possible mechanism of compounds **1** and **2** by examining their regulatory effects on the NF-*κ*B signaling pathway for the first time, demonstrating that these two compounds exert their anti-inflammatory effect by inhibiting the NF-*κ*B signaling pathway. In conclusion, this research provides new scientific evidence for future studies on the anti-inflammatory properties and development of oleanane triterpenoids with C-14 carboxyl group from the *Astilbe* genus.

## 2 Materials and methods

### 2.1 Chemicals, reagents, and antibodies

Compounds **1** and **2** were isolated from *A*. *grandis*. The 14 kg crude powder of *A. grandis* roots was subjected to methanol extraction for four times (3 days/time) to obtain the total extract. The total extract was separated on a macroporous resin column, eluting with water, 50% ethanol and 90% ethanol to obtain 90% ethanol extract. The 90% ethanol extract was subjected to silica gel column chromatography with dichloromethane-methanol (100:1-8:2) gradient elution to obtain three components (A-C). Part B was separated by silica gel column chromatography with petroleum ether-ethyl acetate (100:1-1:1) gradient elution to obtain five components (B1-B5). The B2 fraction was isolated and purified by silica gel column chromatography with dichloromethane-ethyl acetate (100:1-8:2) and Sephadex LH-20 to obtain compounds 1 and 2 (He et al., 2020).

The RAW 264.7 cell line was purchased from Procell Life Science &Technology Co., Ltd. (Wuhan, China). The THP-1 cell line was purchased from Pricella (Wuhan, China). The LPS and Phorbol 12-myristate 13-acetate (PMA) were from Sigma (St. Louis, MO, United States). The fetal bovine serum (FBS) was from Pricella (Wuhan, China). The DMEM was from Servicebio (Wuhan, China). The RPMI-1640 was from Pricella (Wuhan, China). The *β*-mercaptoethanol was from Macklin (Shanghai, China). The Dexamethasone (DEX) was from MedChemExpress (Shanghai, China). The NO detection kit was obtained from Beyotime (Shanghai, China). The CCK8 solution was obtained from TargetMol (Boston, MA, United States). The mouse TNF-*α* ELISA kit, mouse IL-1*β* ELISA kit and mouse IL-6 ELISA kit were obtained from MultiSciences (Hangzhou, China). The human TNF-*α* ELISA kit, human IL-1*β* ELISA kit and human IL-6 ELISA kit were obtained from Neobioscience (Shenzhen, China). The BCA protein concentration determination kit, 5 protein loading buffer and RIPA cell lysate were from Solarbio (Beijing, China). The PAGE gel rapid preparation kit (10%), electrotransfer buffer, electrophoresis buffer, TBST buffer and prestained protein marker Ⅶ (8–195 kDa) were from Servicebio (Wuhan, China). The primary antibody dilution and secondary antibody dilution were from Beyotime (Shanghai, China). The fast sealing liquid was from Genefist (Abingdon, United Kingdom). The polyvinylidene difluoride membranes were from Millipore (Billerica, MA, United States). The antibodies used in the immunoblot analysis, p-p65 and p65 were from Abcam (Cambridge, United Kingdom) while COX-2, iNOS and *β*-actin were from Absin (Shanghai, China). The secondary antibody (anti-rabbit) was from Solarbio (Beijing, China). The luminescent liquid was from Nature Biosciences (New Delhi, India). The total RNA extraction kit was from Solarbio (Beijing, China). The HiScript^®^ II Q RT supermix and SYBR green qPCR master mix kit were from Vazyme (Nanjing, China). The primers for RT-PCR were from Tsingke (Beijing, China). The triton X-100, bovine serum albumin (BSA) and DAPI solution were from Solarbio (Beijing, China). The immunol fluorence staining kit was from Beyotime (Shanghai, China). The tween-80 was from Macklin (Shanghai, China). The 4% paraformaldehyde was from Servicebio (Wuhan, China).

### 2.2 Cell culture

The RAW 264.7 cells were cultured in DMEM supplemented with 1% penicillin/streptomycin, and 10% FBS. Subsequently, the cells were incubated in a 5% CO_2_ atmosphere at 37°C under humidified conditions and sub-cultured every 2 days.

The THP-1 cells were cultured in RPMI-1640 supplemented with 1% penicillin/streptomycin, 10% FBS, and 0.05 mM *β*-mercaptoethanol. Subsequently, the cells were incubated in a 5% CO_2_ atmosphere at 37°C under humidified conditions and sub-cultured every 2 days.

### 2.3 Cell viability

Cell viability was assessed using the CCK8 method. The RAW 264.7 cells were adjusted to a density of 5 × 10^5^ cells/mL, seeded in 96-well plates, and incubated overnight at 37°C with 5% CO_2_ for 20 h. The THP-1 cells were treated with 100 ng/mL PMA, adjusted to a density of 3 × 10^5^ cells/mL, seeded in 96-well plates, and incubated overnight at 37°C with 5% CO_2_ for 48 h to induce their differentiation into macrophages. Subsequently, compounds **1** and **2** were administered at various concentrations (160, 80, 40, 20, 10, and 5 µM). The control group received no drugs and was cultured for 24 h. Finally, 10 µL of CCK8 solution was added to each well and incubated for 1–4 h. Spectrophotometry (Thermo, Rockford, IL, United States) measured the absorbance at 450 nm using a microplate reader, and the relative cell viability was calculated.

### 2.4 Griess method and enzyme-linked immunosorbent assay (ELISA)

To assess the production of NO, TNF-*α*, IL-6, and IL-1*β* in Macrophages, the Griess kit or ELISA kit was utilized. The RAW 264.7 cells were adjusted to a density of 3 × 10^5^ cells/mL, seeded in 6-well plates, and incubated overnight at 37°C with 5% CO_2_ for 20 h. The THP-1 cells were treated with 100 ng/mL PMA, adjusted to a density of 1 × 10^6^ cells/mL, seeded in 6-well plates, and incubated overnight at 37°C with 5% CO_2_ for 48 h. Subsequently, compounds **1** (5, 10, and 20 μM), compounds **2** (20, 40, and 80 μM) and 1 μM DEX (Jeon et al., 2000) were added for 1 h, followed by stimulation with 1 μg/mL LPS (Montoya-Rodríguez et al., 2014) for an additional 20 h. After incubation, the cell supernatant was collected. The NO concentration in the cell supernatant was detected using Griess kit, and the concentrations of TNF-*α*, IL-6, and IL-1*β* in the cell supernatant were determined using the ELISA kit according to the provided instructions.

### 2.5 Western blotting

The expression of COX-2, iNOS and p-p65 proteins was assessed using Western blotting. The RAW 264.7 cells were adjusted to a density of 5 × 10^5^ cells/mL, seeded in 6-well plates, and incubated overnight at 37°C with 5% CO_2_ for 20 h. The THP-1 cells were treated with 100 ng/mL PMA, adjusted to a density of 1 × 10^6^ cells/mL, seeded in 6-well plates, and incubated overnight at 37°C with 5% CO_2_ for 48 h. Following incubation, compounds **1** (5, 10, and 20 μM), compounds **2** (20, 40, and 80 μM) and DEX (1 μM) administered for 1 h, followed by stimulation with 1 μg/mL LPS for an additional 20 h. Specifically for detecting the expression of p-p65, the RAW 264.7 cell density was adjusted to 5 × 10^5^ cells/mL, and the cells were treated with drugs for 1 h, followed by stimulation with 1 μg/mL LPS for 30 min. After incubation, total cell protein was extracted, protein quantified using the BCA protein assay kit, and the proteins were denatured for western blotting.

Proteins from each sample were separated by 10% sodium dodecyl sulfate-polyacrylamide gel electrophoresis (SDS-PAGE) and then transferred to polyvinylidene difluoride membranes (PVDF). Subsequently, the membranes were blocked in a fast-sealing liquid and incubated overnight at 4°C with antibodies against iNOS (1:1,000), COX-2 (1:3,000), p-p65 (1:1,000), p65 (1:1,000) and *β*-actin (1:1,000). Following this, the membranes underwent incubation with horseradish peroxidase (HRP)-coupled secondary antibodies (1:1,000) for 1 h at room temperature. Immunoreactive protein bands were detected using an ECL reagent and the ChemiDoc Imaging System (Bio-Rad, Hercules, California, United States).

### 2.6 Quantitative real-time PCR (qRT-PCR)

The RAW 264.7 cells were adjusted to a density of 5 × 10^5^ cells/mL, seeded in 6-well plates, and incubated overnight at 37°C with 5% CO_2_ for 20 h. The THP-1 cells were treated with 100 ng/mL PMA, adjusted to a density of 1 × 10^6^ cells/mL, seeded in 6-well plates, and incubated overnight at 37°C with 5% CO_2_ for 48 h. Following this, compounds **1** (5, 10, 20 μM), compounds **2** (20, 40, and 80 μM) and DEX (1 μM) were administered for 1 h, then stimulation with 1 μg/mL LPS for an additional 20 h. After incubation, total RNA was isolated using a total RNA extraction kit. The isolated total RNA (1 μg) was reverse transcribed into cDNA using the HiScript^®^ II Q RT SuperMix. Real-time quantitative PCR was performed by using the SYBR Green qPCR Master Mix kit according to the provided instructions. The PCR program comprised one cycle at 95°C for 10 min; 40 cycles of 95°C for 15 s and 60°C for 60 s. Quantification was performed using the 2^(−△△Ct)^ method, and data were normalized to the expression of *β*-actin.

### 2.7 Immunofluorescence staining

The RAW 264.7 cells were adjusted to a density of 2 × 10^5^ cells/mL, seeded in 6-well plates with cell climbing slices, and incubated overnight at 37°C with 5% CO_2_ for 24 h. Overnight, the cells were pre-treated with compounds **1** and **2** for 1 h and then stimulated with LPS (1 μg/mL) for 30 min. Next, cells were washed once with PBS, fixed with 4% paraformaldehyde for 20 min, washed three times with PBS, permeabilized with PBS containing 0.5% triton X-100 for 15 min, washed three times with PBS, and blocked with 5% BSA (containing 0.3% triton X-100) for 1 h at room temperature. Subsequently, the cells were incubated with the primary antibody against NF-*κ*B p65 (1:250) overnight at 4°C, washed four times with a washing solution (PBS containing 1% tween-80), and then incubated with the secondary antibody for 1 h at room temperature. After four times washes with the washing solution, the cells were stained with 4′,6-diamidino-2-phenylindole (DAPI) for 20 min, washed three times with PBS, and then observed using a fluorescence microscope (IX73, Olympus, Tokyo, Japan).

### 2.8 Molecular docking simulation

The crystal structure of NF-*κ*B p65 (PDB ID: 6NV2) was obtained from the Protein Data Bank (https://www.rcsb.org/). The three-dimensional structures of compounds **1** and **2** were generated using ChemDraw 19.0. Subsequently, both structures and the protein structure were imported into CB-DOCK2 (https://cadd.labshare.cn/cb-dock2/php/index.php), which was used to assessing the potential interactions between the candidate compounds and the target protein (NF-*κ*B p65).

### 2.9 Statistic analysis

All values in the figures and text in this paper are expressed as mean ± SD (n = 3). Data were analyzed using GraphPad Prism 9.5.1. Statistical analysis employed one-way ANOVA to assess significant differences between groups, followed by Tukey’s multiple comparison test to calculate differences between groups. A *P*-value less than 0.05 was considered statistically significant.

## 3 Results

### 3.1 Effects of compounds 1 and 2 on cell viability in RAW 264.7 cells and THP-1 cells

**FIGURE 1:**
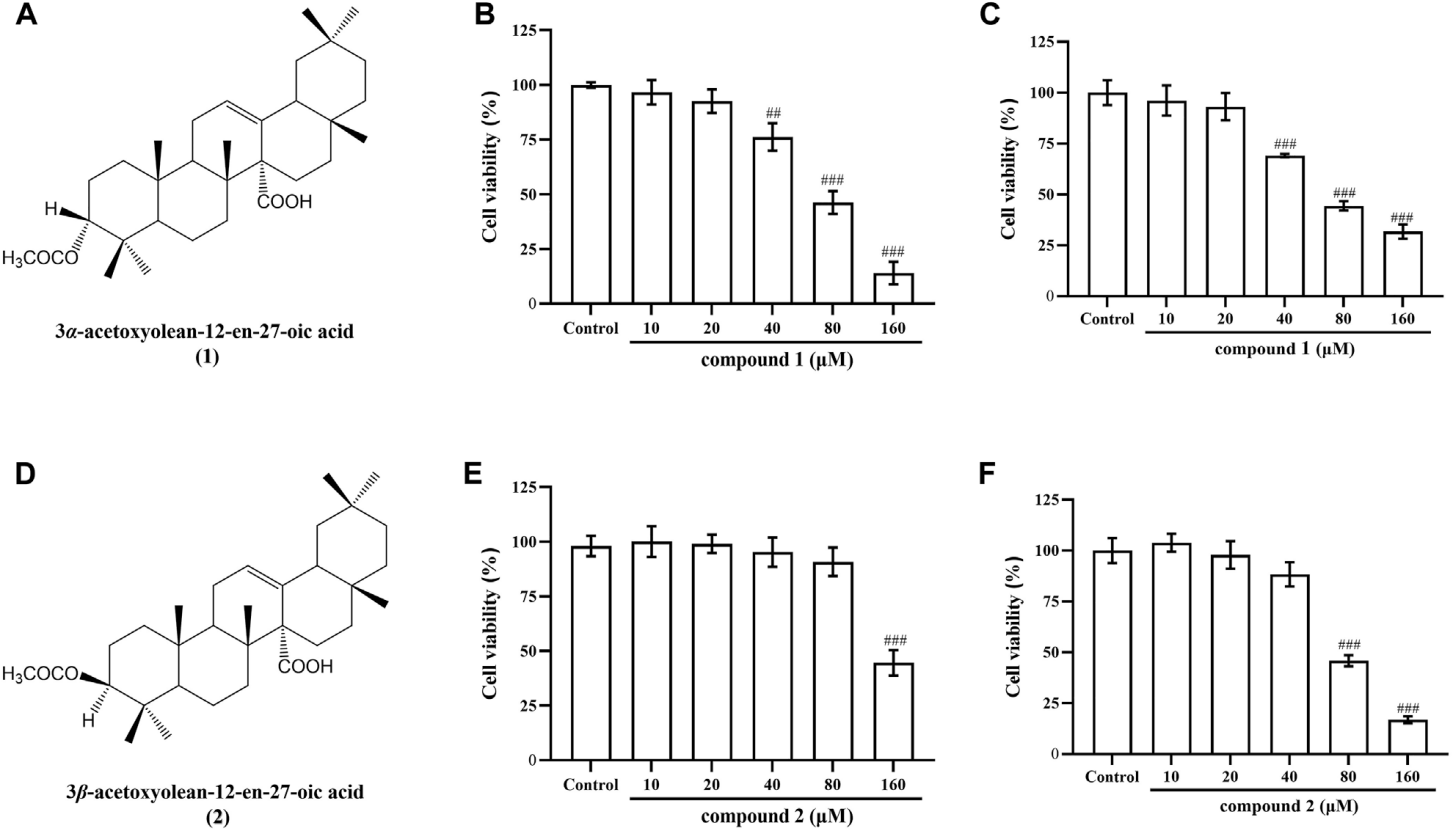
Chemical structural formula of compounds **1** and **2**, and their effects on the cell viability in RAW 264.7 cells and THP-1 cells. **(A)** Chemical structural formula of compound **1**. **(B)** Effect of compound **1** on the cell viability of RAW 264.7 cells. **(C)** Effect of compound **1** on the cell viability of THP-1 cells. **(D)** Chemical structural formula of compound **2**. **(E)** Effect of compound **2** on the cell viability of RAW 264.7 cells. **(F)** Effect of compound **2** on the cell viability of THP-1 cells. Data are presented as mean ± SD. ^*###*^ *P* < 0.001 vs. Control group, ^*##*^ *P* < 0.01 vs. Control group.

To determine the safe therapeutic concentration of compounds, this study utilized the CCK8 method to screen drugs concentrations. Chemical structural formula of compound 1 was showed in Figure 1A and Chemical structural formula of compound 2 was showed in Figure 1D. It was observed that compound **1** did not affect the cell viability of RAW 264.7 cells and THP-1 cells at concentrations of 5, 10 and 20 μM (Figures 1B, C). Similarly, compound **2** did not impact the cell viability of RAW 264.7 cells at concentrations of 20, 40, and 80 μM (Figure 1E) and did not impact the cell viability of THP-1 cells at concentrations of 10, 20 and 40 μM (Figure 1F). Consequently, these concentrations were selected for use in subsequent experiments.

### 3.2 Effects of compounds 1 and 2 on NO production in RAW 264.7 cells

**FIGURE 2:**
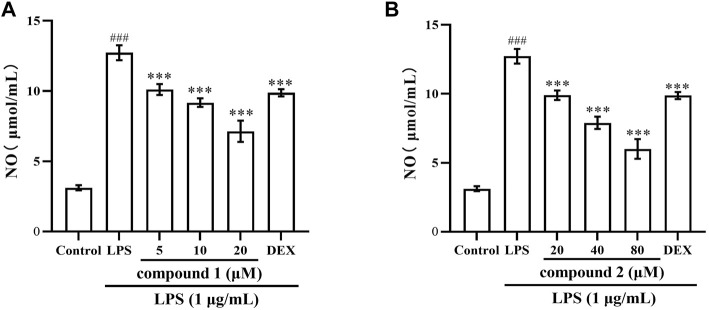
Compounds **1** and **2** effects on LPS-induced NO production in RAW 264.7 cells. **(A)** NO secretion in LPS-induced RAW 264.7 cells after pretreatment with compound **1**. **(B)** NO secretion in LPS-induced RAW 264.7 cells after pretreatment with compound **2**. Data are presented as mean ± SD. ^*###*^ *P* < 0.001 vs. Control group, ***P < 0.001 vs. LPS group.

In LPS-induced RAW 264.7 cells, the secretion of NO serves as a marker indicating the onset of inflammation (Luo et al., 2018). As illustrated in Figure 2, When the cells were exposed to LPS for 20 h, the production of NO in the cell supernatant was significantly increased (*P* < 0.001). Pretreatment with compound **1** or **2** for 1 h obviously and concentration-dependently reduced the NO production induced by LPS in LPS-stimulated RAW 264.7 cells (*P* < 0.001). DEX is a classic antiinflammatory drug that significantly inhibits the release of inflammatory indicators (Kim et al., 2014), therefore, it was selected as a positive control to evaluate the anti-inflammatory activity of compound **1** and **2** in this study. The results indicate that DEX significantly reduced NO levels (*P* < 0.01), while the mid and high concentration groups of compounds 1 and 2 demonstrated more significant effects compared to DEX.

### 3.3 Effects of compounds 1 and 2 on LPS-induced TNF-*α*, IL-6 and IL-1*β* levels in RAW 264.7 cells and THP-1 cells

**FIGURE 3:**
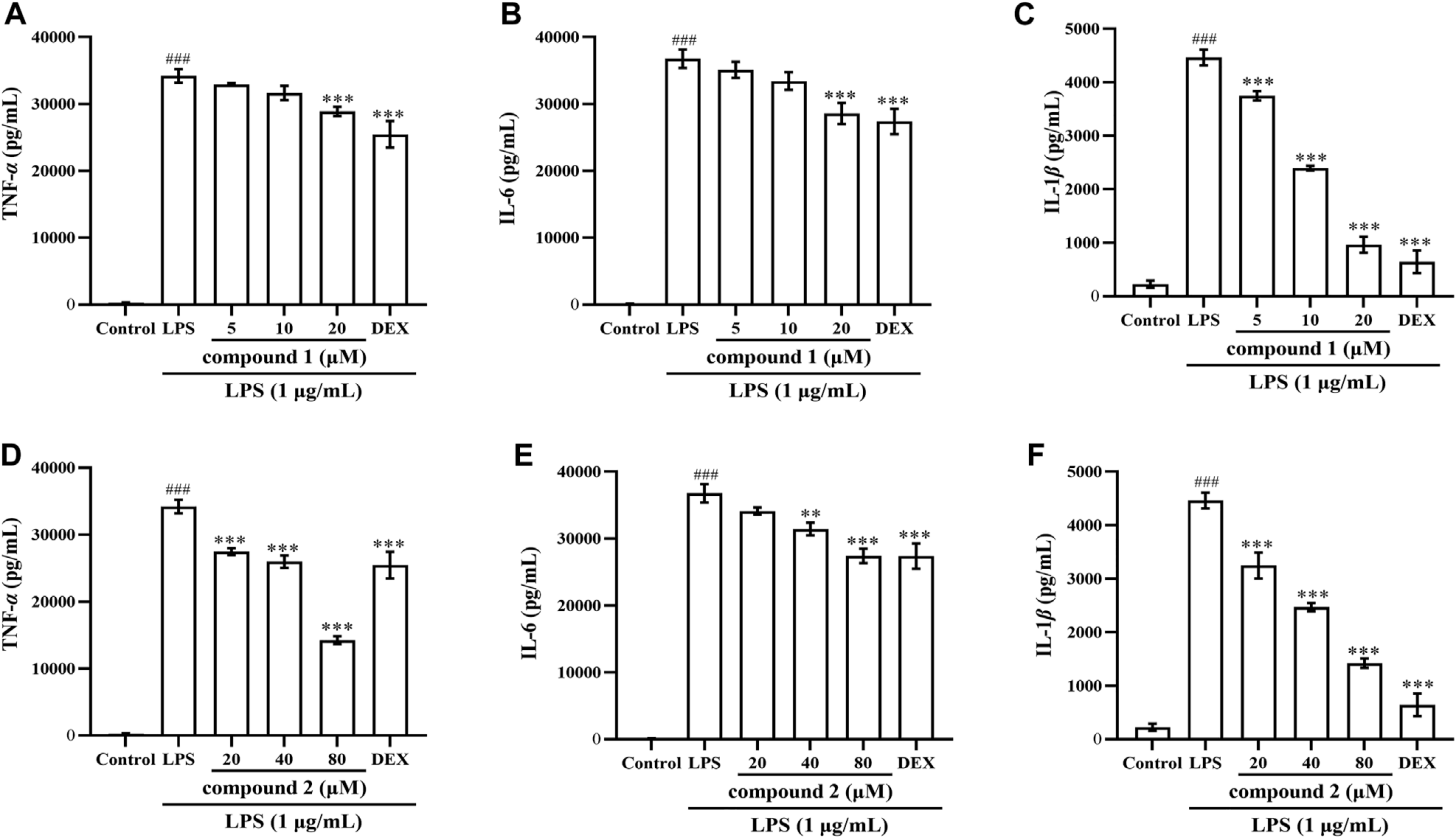
Effects of compounds **1** and **2** on LPS-induced proinflammatory cytokine production in RAW 264.7 cells. **(A)** TNF-*α* in the cell supernatant pretreated with compound **1**. **(B)** IL-6 in the cell supernatant pretreated with compound **1**. **(C)** IL-1*β* in the cell supernatant pretreated with compound **1**. **(D)** TNF-*α* in the cell supernatant pretreated with compound **2**. **(E)** IL-6 in the cell supernatant pretreated with compound **2**. **(F)** IL-1β*β* in the cell supernatant pretreated with compound **2**. Data are presented as mean ± SD. ^*###*^ *P* < 0.001 vs. Control group, **P < 0.01, ***P < 0.001 vs. LPS group.

**FIGURE 4:**
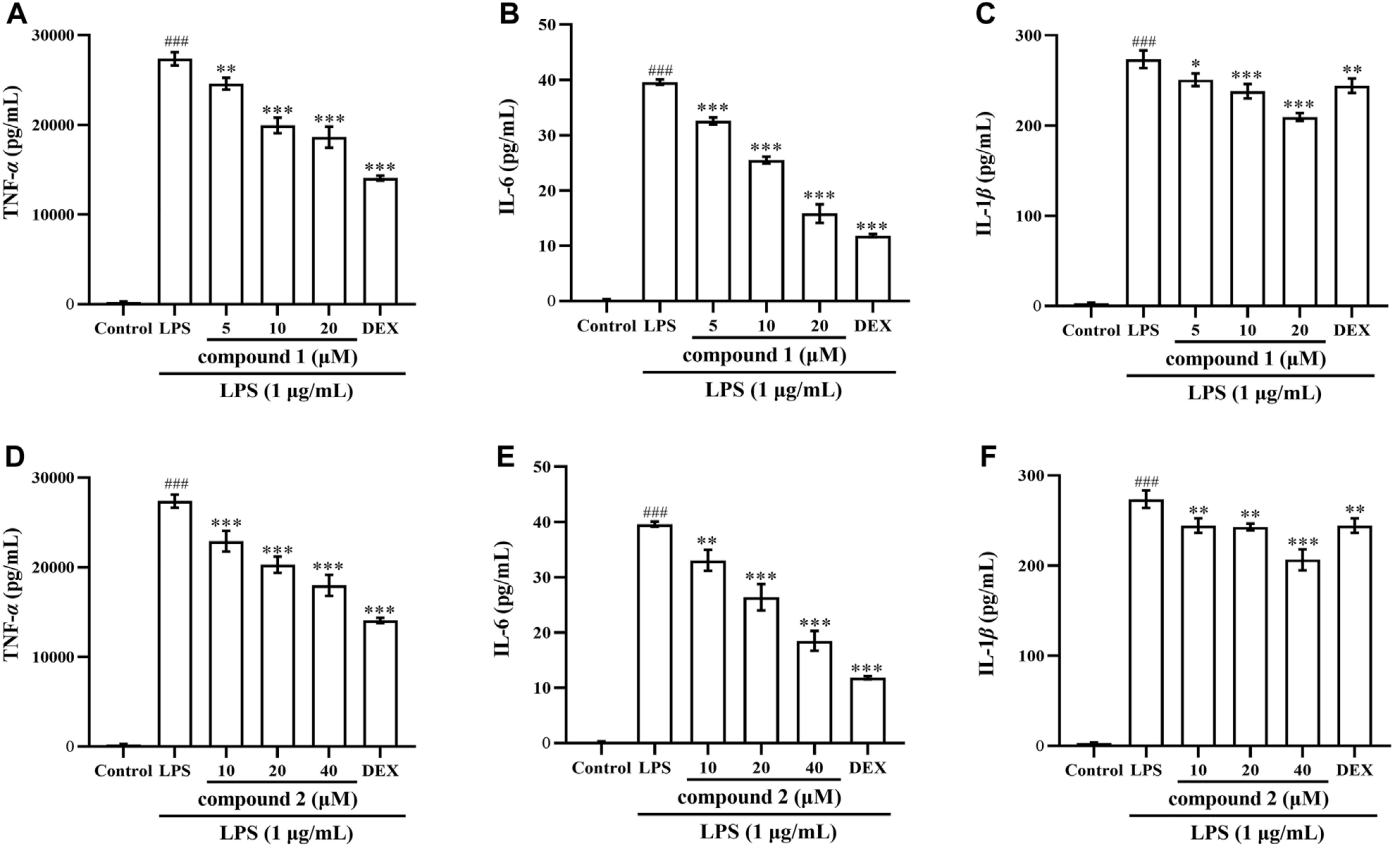
Effects of compounds **1** and **2** on LPS-induced proinflammatory cytokine production in THP-1 cells. **(A)** TNF-α in the cell supernatant pretreated with compound **1**. **(B)** IL-6 in the cell supernatant pretreated with compound **1**. **(C)** IL-1β in the cell supernatant pretreated with compound **1**. **(D)** TNF-α in the cell supernatant pretreated with compound **2**. **(E)** IL-6 in the cell supernatant pretreated with compound **2**. **(F)** IL-1β in the cell supernatant pretreated with compound **2**. Data are presented as mean ± SD. ^*###*^ *P* < 0.001 vs. Control group, *P < 0.05, **P < 0.01, ***P < 0.001 vs. LPS group.

To further validate the anti-inflammatory effects of compounds **1** and **2** in LPS-induced RAW 264.7 cells and THP-1 cells, we assessed the expression of TNF-*α*, IL-6 and IL-1*β* in cell supernatant following pretreatment with these two compounds. As depicted in Figures 3, 4, the levels of TNF-*α*, IL-6 and IL-1*β* in LPS-induced cells were significantly elevated compared to the control group (*P* < 0.001); however, pretreatment with compound **1**, compound **2**, and DEX resulted in varying degrees of decrease in the levels of these proinflammatory cytokines.

### 3.4 Effects of compounds 1 and 2 on iNOS and COX-2 protein expression in LPS-induced RAW 264.7 cells

**FIGURE 5:**
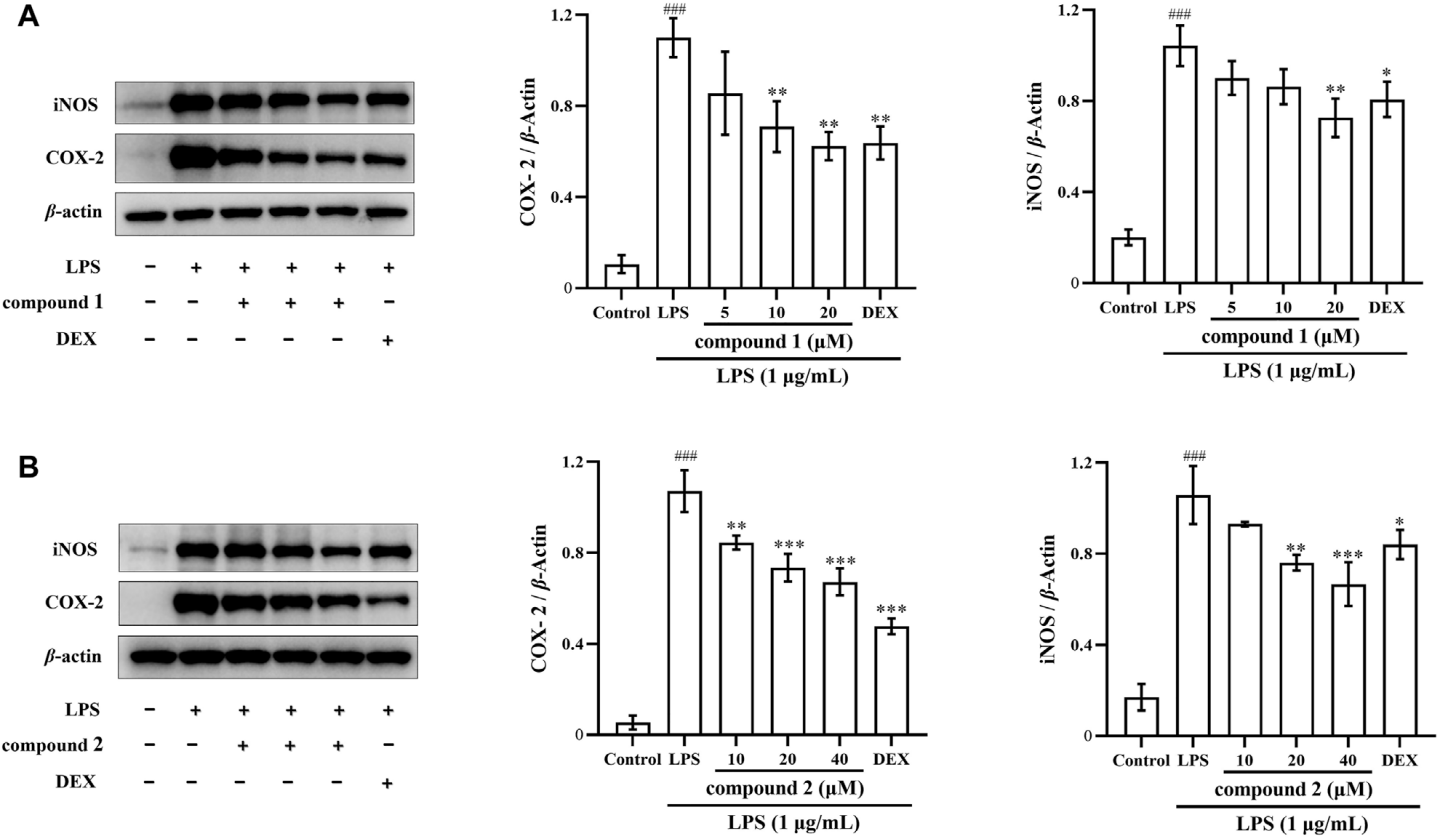
Effects of compounds **1** and **2** on LPS-induced iNOS and COX-2 protein expression in RAW 264.7 cells. **(A)** Expression of iNOS and COX-2 in LPS-induced RAW 264.7 cells pretreated with compound **1**. **(B)** Expression of iNOS and COX-2 in LPS-induced RAW 264.7 cells pretreated with compound **2**. Data are presented as mean ± SD. ^*###*^ *P* < 0.001 vs. Control group, *P < 0.05, **P < 0.01, ***P < 0.001 vs. LPS group.

Next, we investigated whether the inhibitory effects of compounds **1** and **2** on NO and proinflammatory cytokine production are related to the regulation of iNOS and COX-2 expression using western blotting. In Figures 5A, B, the expression of iNOS and COX-2 proteins significantly increased in LPS-induced RAW 264.7 cells compared to the control group (*P* < 0.001); however, the protein expression of iNOS and COX-2 significantly decreased in a concentration-dependent manner after pretreatment with compound **1**, compound **2**, and DEX. Furthermore, the high concentration groups of both compounds showed more pronounced inhibition of iNOS protein expression compared to the DEX group.

### 3.5 Effects of compounds 1 and 2 on COX-2 protein expression in LPS-induced THP-1 cells

**FIGURE 6:**
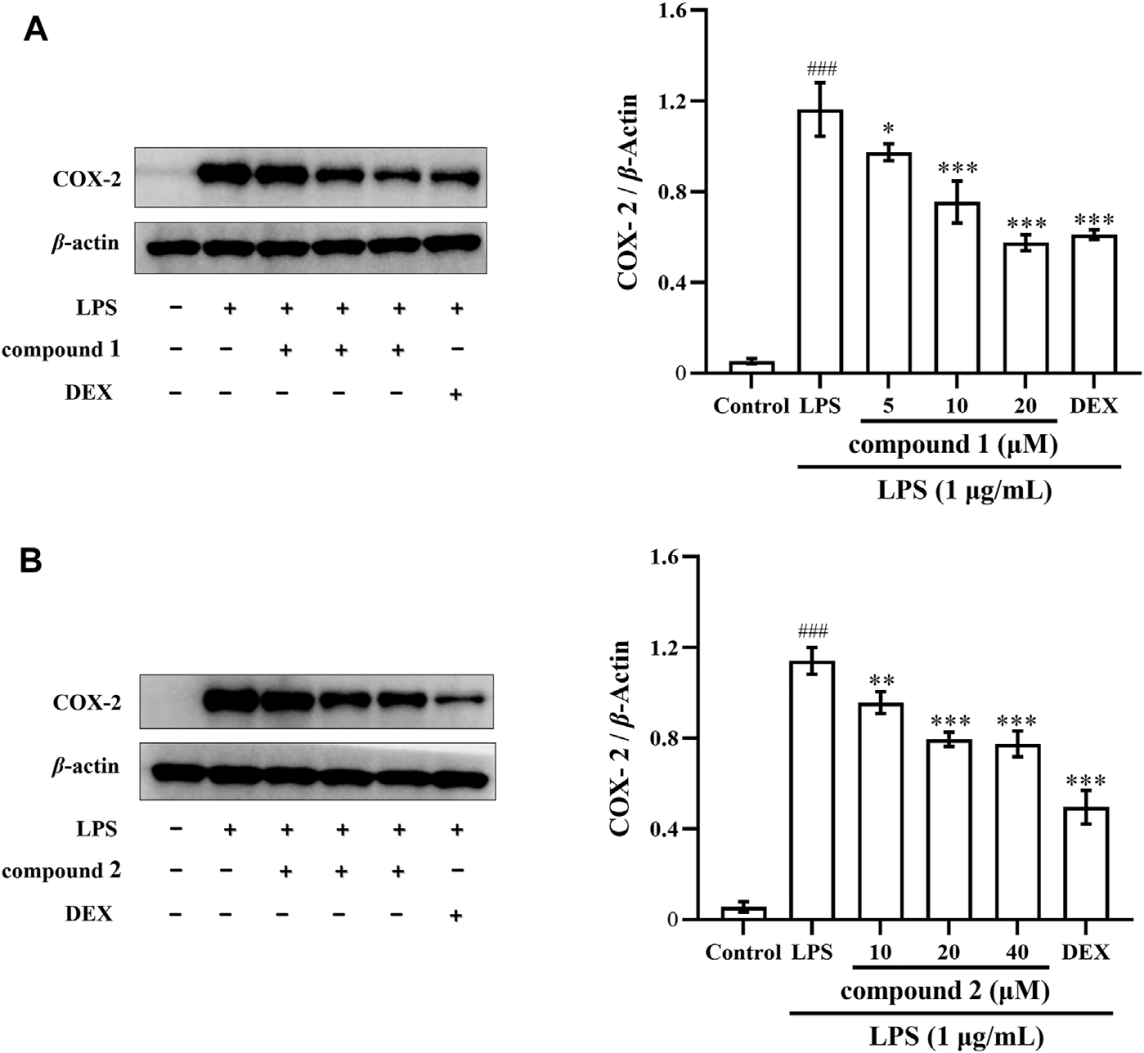
Effects of compounds **1** and **2** on LPS-induced COX-2 protein expression in THP-1 cells. **(A)** Expression of COX-2 in LPS-induced THP-1 cells pretreated with compound **1**. **(B)** Expression of COX-2 in LPS-induced THP-1 cells pretreated with compound **2**. Data are presented as mean ± SD. ^*###*^ *P* < 0.001 vs. Control group, *P < 0.05, **P < 0.01, ***P < 0.001 vs. LPS group.

The expression of the inflammatory cytokine COX-2 was significantly increased in the LPS-induced THP-1 cells (Luo et al., 2018). As illustrated in Figures 6A, B, the expression of COX-2 proteins significantly increased in LPS-induced THP-1 cells compared to the control group (*P* < 0.001); however, the protein expression of COX-2 significantly decreased in a concentration-dependent manner after pretreatment with compound **1** and compound **2**, positive drug DEX also significantly decreased the protein expression of COX-2.

### 3.6 Effects of compounds 1 and 2 on iNOS and COX-2 mRNA expression in LPS-induced RAW 264.7 cells and THP-1 cells

**FIGURE 7:**
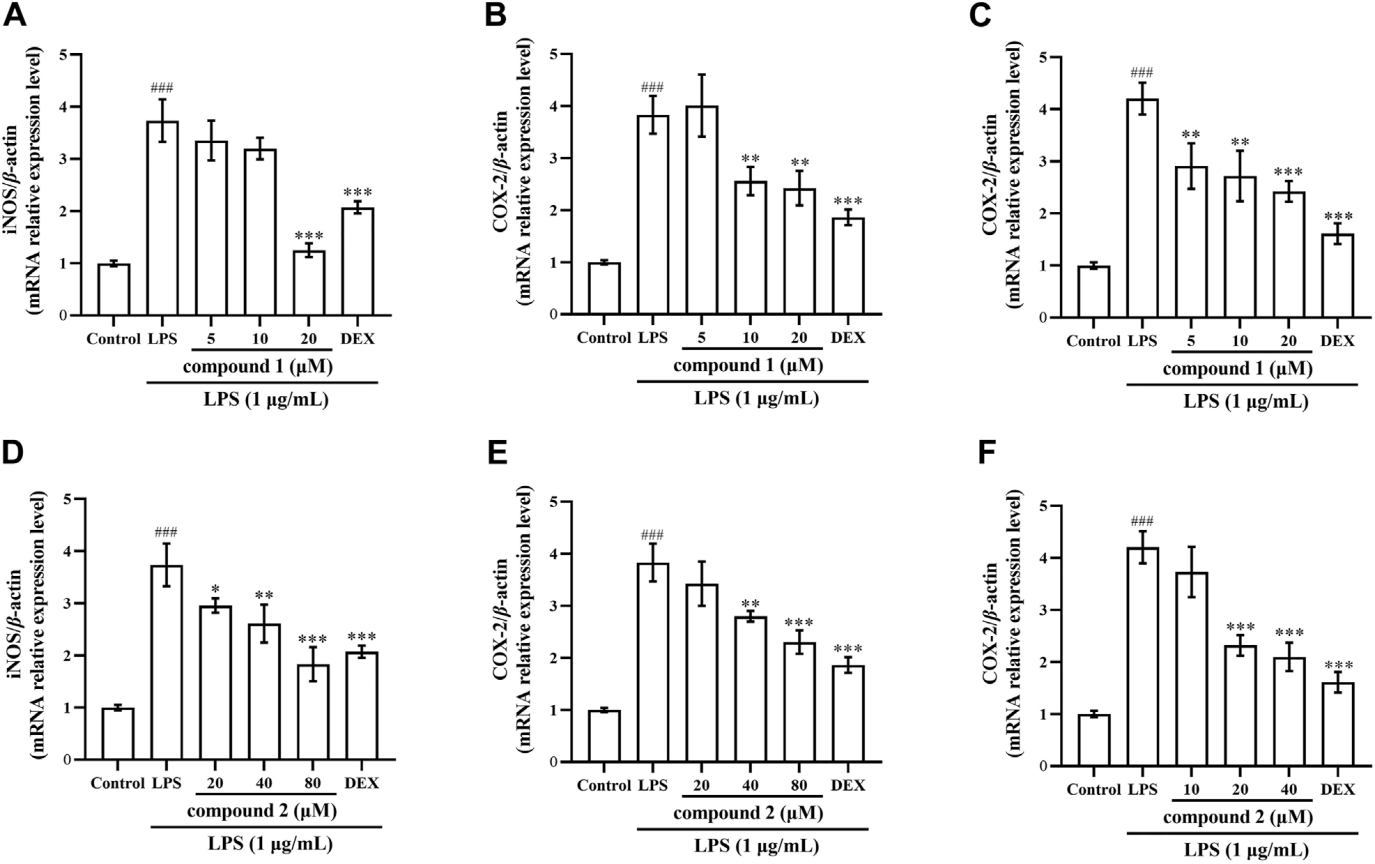
Effects of compounds **1** and **2** on LPS-induced iNOS and COX-2 mRNA expression in RAW 264.7 cells and THP-1 cells. **(A)** iNOS mRNA expression in RAW 264.7 cells after pretreatment with compound **1**. **(B)** COX-2 mRNA expression in RAW 264.7 cells pretreated with compound **1**. **(C)** COX-2 mRNA expression in THP-1 cells pretreated with compound **1**. **(D)** iNOS mRNA expression in RAW 264.7 cells pretreated with compound **2**. **(E)** COX-2 mRNA expression in RAW 264.7 cells pretreated with compound **2**. **(F)** COX-2 mRNA expression in THP-1 cells pretreated with compound **2**. Data are presented as the mean ± SD. ^*###*^ *P* < 0.001 vs. Control group, *P < 0.05, **P < 0.01, ***P < 0.001 vs. LPS group.

Next, we verified the inhibitory effects of compounds **1** and **2** on iNOS and COX-2 expression using qRT-PCR. In Figures 7A, B, D, E, both iNOS and COX-2 mRNA expression significantly increased in RAW 264.7 cells exposed to LPS compared with the control group (*P* < 0.001); however, pretreatment with compound **1**, compound **2**, and DEX led to a significant concentration-dependent decrease both in iNOS and COX-2 mRNA expression compared with the LPS group. In Figures 7C, F, COX-2 mRNA expression significantly increased in THP-1 cells exposed to LPS compared with the control group (*P* < 0.001); however, pretreatment with drugs significantly decreased COX-2 mRNA expression compared to the LPS group.

### 3.7 Effects of compounds 1 and 2 on NF-*κ*B activation in LPS-induced RAW 264.7 cells

**FIGURE 8:**
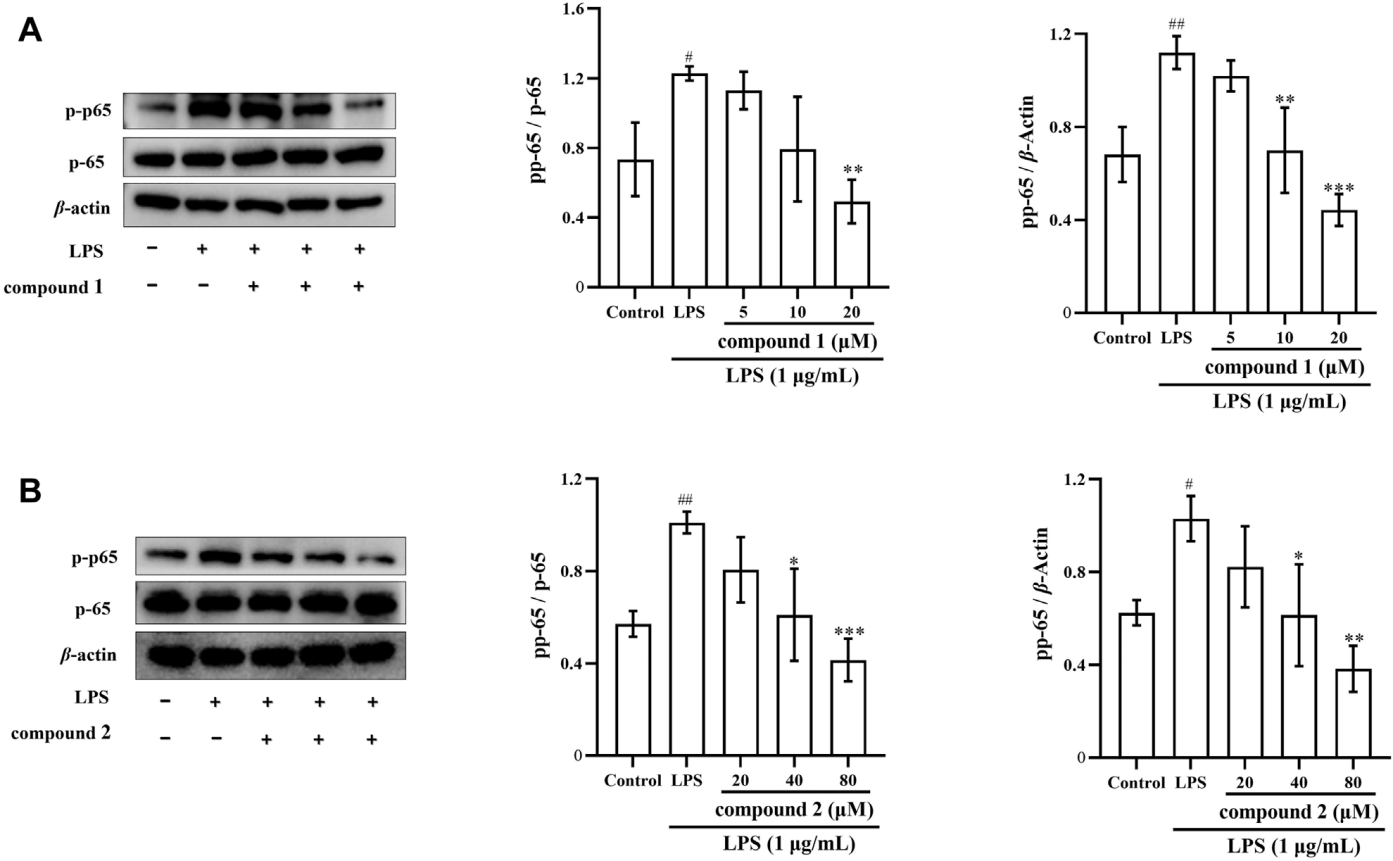
The pharmacological action of compounds **1** and **2** on the levels of p-p65 in LPS-induced RAW 264.7 cells. **(A)** Expression of p-p65 after compound **1** pretreatment. **(B)** Expression of p-p65 after pretreatment with compound **2**. Data are presented as mean ± SD. ^*#*^ *P* < 0.05, ^*##*^ *P* < 0.01, ^*###*^ *P* < 0.001 vs. Control group, *P < 0.05, **P < 0.01, ***P < 0.001 vs. LPS group.

To further explore the anti-inflammatory mechanism of compounds **1** and **2**, we investigated whether these compounds inhibit the phosphorylation of p65 in LPS-induced RAW 264.7 cells. We observed that LPS significantly increased p65 protein phosphorylation in RAW 264.7 cells; however, this phosphorylation was significantly inhibited after pretreatment with compounds **1** and **2** (Figures 8A, B).

### 3.8 Effects of compounds 1 and 2 on LPS-induced nuclear translocation of NF-*κ*B p65

**FIGURE 9:**
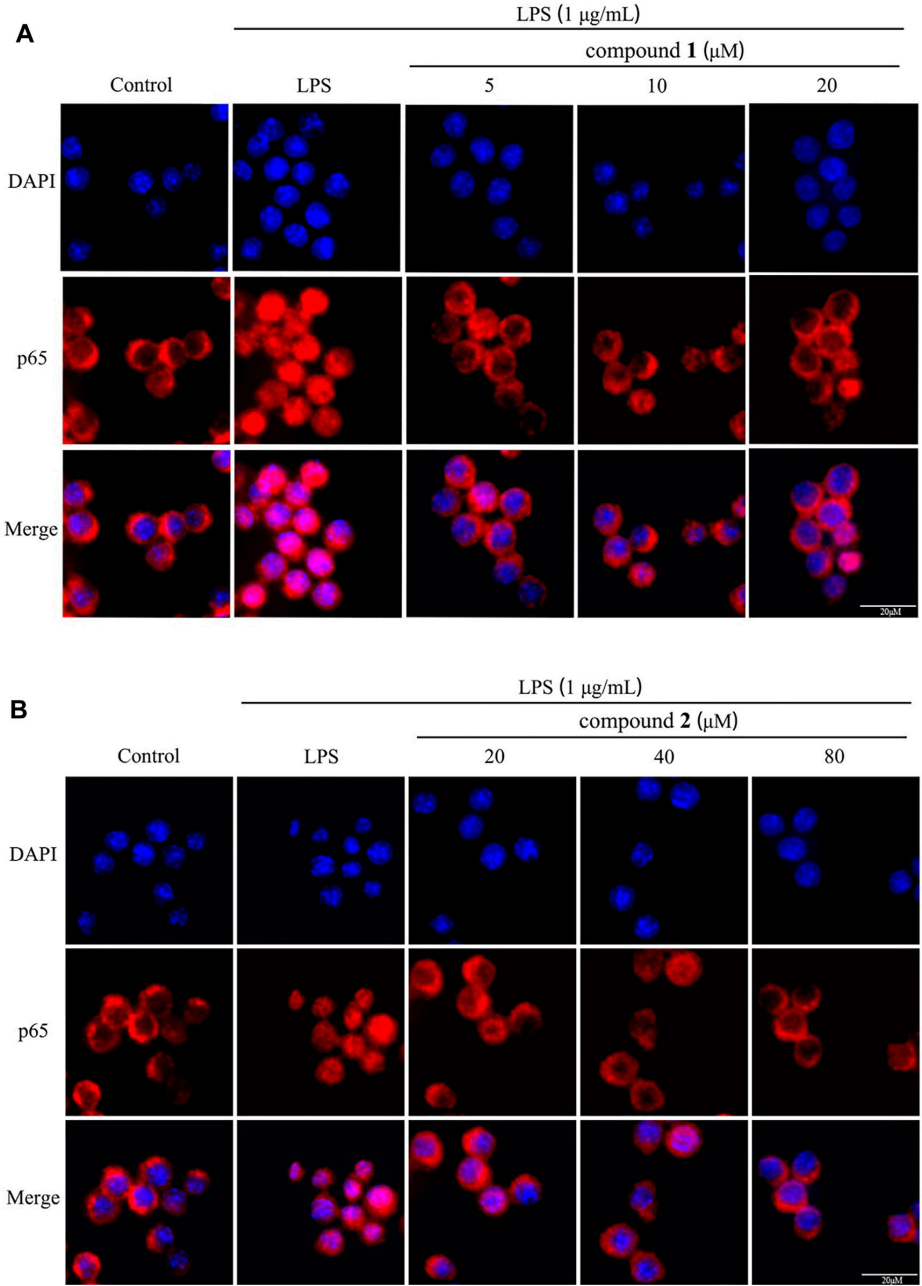
Effects of compounds **1** and **2** on LPS-induced nuclear translocation of NF-*κ*B p65 in RAW 264.7 cells. **(A)** The nuclear translocation of NF-*κ*B p65 after pretreatment with compound **1** (n = 3). **(B)** The nuclear translocation of NF-*κ*B p65 pretreated with compound **2** (n = 3).

To assess the impact of compounds **1** and **2** on the NF-*κ*B pathway, we examined the nuclear levels of NF-*κ*B p65 using immunofluorescence staining. As illustrated in Figures 9A, B, LPS stimulation induced the translocation of NF-*κ*B p65 from the cytosol to the nucleus; however, pretreatment with compounds **1** and **2** effectively mitigated the nuclear translocation of NF-*κ*B p65 in a concentration-dependent manner.

### 3.9 Molecular docking results for compounds 1 and 2 with target protein (NF-*κ*B p65)

**FIGURE 10:**
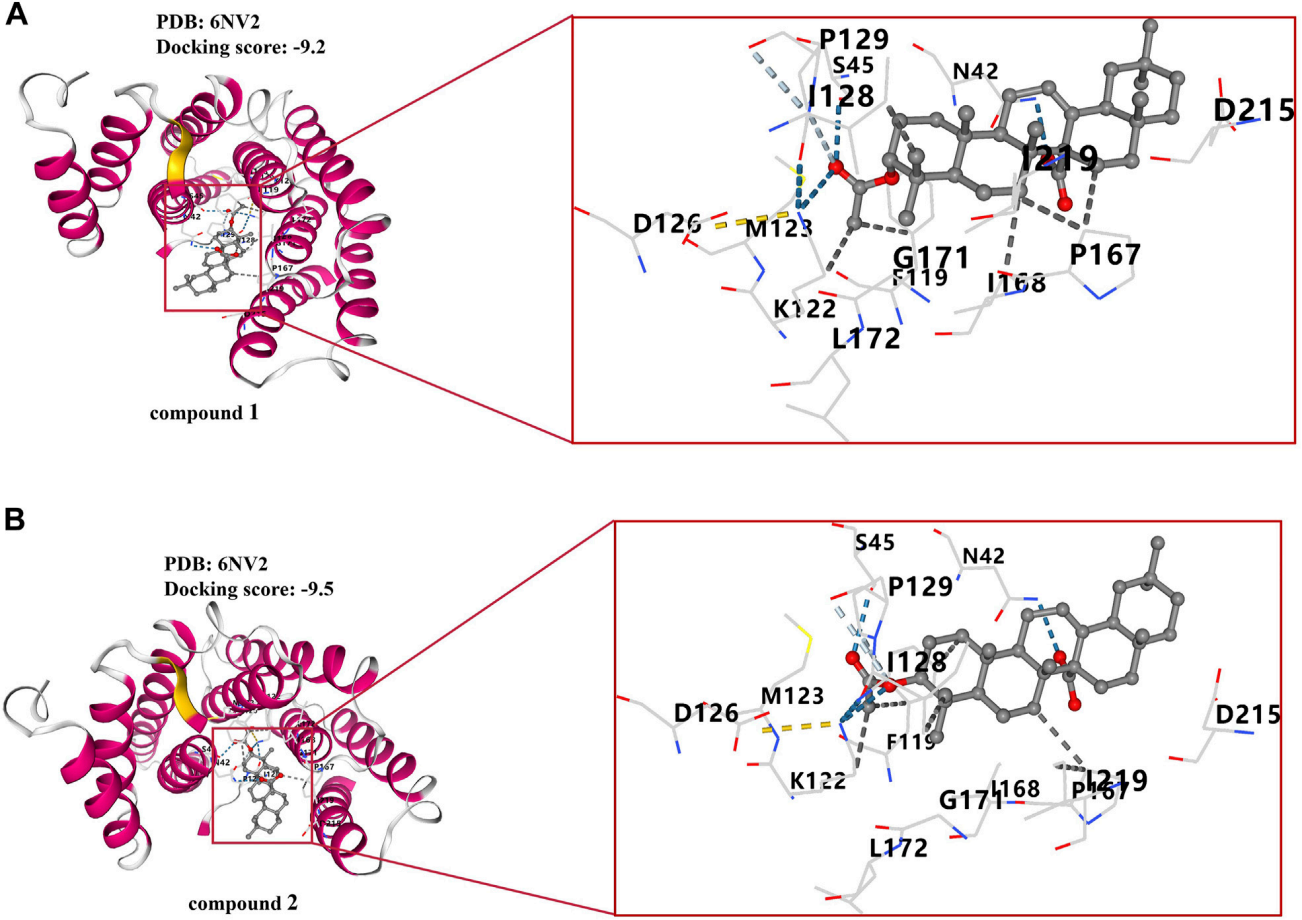
Molecular docking results of compounds **1** and **2**. **(A)** Docking results of compound **1** with NF-*κ*B p65 complex. **(B)** Docking results of compound **2** with NF-*κ*B p65 complex.

To further validate the relevance of the compounds to the NF-κB signaling pathway, we conducted reverse validation through molecular docking. Molecular docking assays revealed that both compounds **1** and **2** were situated within the pocket of the NF-*κ*B p65 (6NV2) complex, as illustrated in Figures 10A, B. Additionally, these compounds interacted with multiple amino acid sites, as depicted in the same figure. The molecular docking score for compound **1** was found to be −9.2, while the molecular docking score for compound **2** was −9.5.

## 4 Discussion

Currently, unbridled inflammation poses an escalating threat to both the physical and mental wellbeing of humans. Prolonged inflammatory reactions, stemming from systemic inflammatory response syndrome, autoimmune diseases, and numerous chronic conditions is often accompanied by human life. Furthermore, the incidence of cancer-related deaths attributable to inflammation is not insignificant (Hussain and Harris, 2007). Consequently, there is an imperative need to explore and develop highly effective anti-inflammatory drugs.

Miao medicines have a history spanning thousands of years in treating inflammatory diseases, featuring abundant resources, remarkable medicinal efficacy, and minimal toxic side effects. Despite these virtues, most Miao medicines remain underexplored, with limited research, and the active ingredients, along with their mechanisms of action, often remain unknown (Liu et al., 2017). For instance, *A*. *grandis* is commonly used in the folk, exhibiting significant anti-inflammatory and analgesic effects. However, the utilization of *A*. *grandis* remains largely empirical, with limited studies on the anti-inflammatory activity of its active ingredients (Jiang et al., 2006; Wang, et al., 2013; He et al., 2020; He et al., 2021). Notably, the anti-inflammatory potential of its characteristic oleanane triterpenoids with C-14 carboxyl group has been understudied. Given the contemporary demand for anti-inflammatory treatments, a primary objective is to explore active compounds in Miao medical resources for anti-inflammatory research. In this study, we pioneered the establishment of a classical model using LPS-induced RAW 264.7 cells and THP-1 cells for the preliminary exploration of the anti-inflammatory activities and mechanism of characteristic oleanane triterpenoids with C-14 carboxyl group (compounds **1** and **2**), isolated from the Miao medicine *A*. *grandis*. The purpose of this study is to establish a foundation for the clinical application of *A*. *grandis* and provide scientific data support for further research on the anti-inflammatory activity of oleanane triterpenoids with C-14 carboxyl group.

Macrophages serve as the key arbiters of the innate immune system, constituting one of the primary lines of defense against invading pathogens and playing a vital role in the body’s immune system (Maione et al., 2017; Keewan and Naser, 2020). LPS, a major product of Gram-negative bacteria, has the capacity to stimulate the activation of immune cells, particularly macrophages, triggering a cascade of systemic inflammation and release of inflammatory factors (Rossol et al., 2011). Among these factors, IL-6 and IL-1*β* play pivotal roles in activating macrophages and contribute to both acute and chronic inflammation. TNF-*α*, functioning as a macrophage activator and initiator of the immune response, holds a central position in the inflammatory cascade. All these inflammatory factors are stimulated by external factors and interact with each other to collectively manage the inflammatory response and participate in the disease process (Xie et al., 2012; Sampaio et al., 2013). Therefore, our investigation aimed to examine whether compounds **1** and **2** suppressed these cytokines in LPS-induced RAW 264.7 cells and THP-1 cells. The results demonstrated that both compounds **1** and **2** significantly inhibited the expression of IL-6, IL-1*β* and TNF-*α* in the cell supernatant (Figures 3, 4).

NO serves as a key mediator during the inflammatory response, with iNOS producing substantial amounts of NO in macrophages after exposure to LPS (Rahman et al., 2021; Luo et al., 2022). Selective inhibition of iNOS has been shown to ameliorate LPS-induced organ damage (Feihl et al., 2001). In addition, studies have shown that high levels of COX-2 expression can lead to decreased expression of immune cells and increase the risk of inflammatory diseases (Cui and Jia, 2021). Therefore, our investigation aimed to determine whether compounds **1** and **2** inhibited iNOS and NO, as well as inhibited COX-2 expression. The results indicated that these two compounds reduced NO and iNOS expression in LPS-induced RAW 264.7 cells (Figures 2, 5, 7) and also inhibited COX-2 protein and mRNA expression in both LPS-induced RAW 264.7 cells and THP-1 cells (Figures 5–7).

**FIGURE 11:**
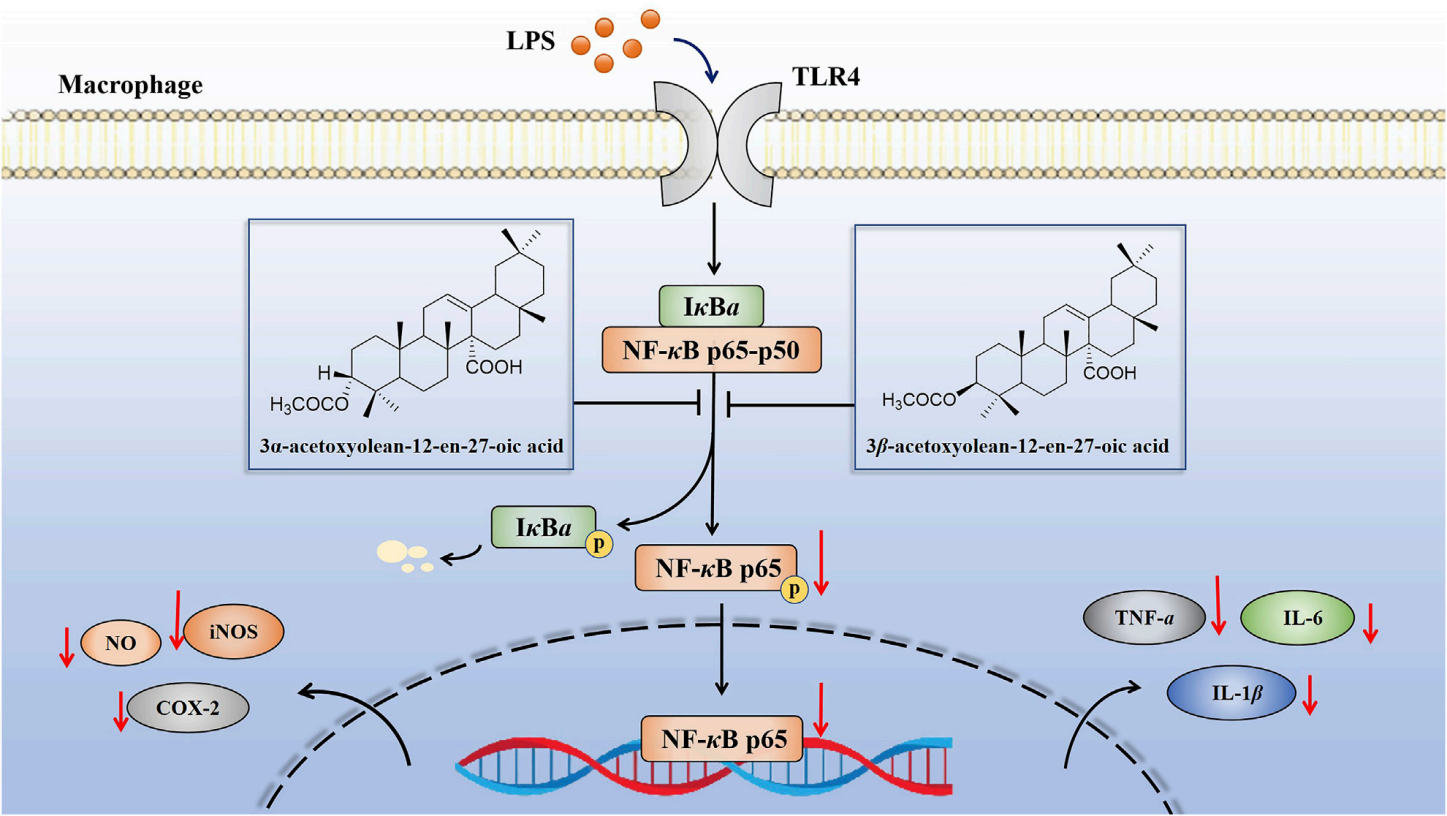
Schematic of the anti-inflammatory activities of compounds **1** and **2** in LPS-induced RAW 264.7 cells. These compounds exhibited anti-inflammatory activity by inhibiting the expression of inflammatory mediators (NO, TNF-*α*, IL-6 and IL-1*β*) and inflammatory related proteins (iNOS and COX-2) through the suppression of the NF-*κ*B pathway.

Prior studies have demonstrated that, both iNOS and COX-2 belong to the downstream indicators of the NF-*κ*B pathway (Han et al., 2004). NF-*κ*B serves as the initiator for the transcription of inflammatory genes such as TNF-*α*, IL-6, IL-1*β*, COX-2 and iNOS (Figure 11) (Won et al., 2006; Li et al., 2020). Inhibiting the activation of the NF-*κ*B pathway is recognized as an effective strategy to mitigate the release of inflammatory cytokines, reduce the expression of inflammatory proteins, and control the development and progression of inflammatory diseases (Han et al., 2019; Notarte et al., 2023). In this experiment, further tests on key signaling pathway proteins (p-p65 and p65) of the NF-*κ*B pathway were conducted to confirm whether compounds **1** and **2** exerted anti-inflammatory effects by inhibiting the activation of the NF-*κ*B pathway. Our findings indicated that these two compounds inhibited the activation of the NF-*κ*B pathway by attenuating the phosphorylation of the p65 protein and NF-*κ*B p65 translocation, thereby suppressed the expression of proinflammatory cytokines and mediators (Figures 8, 9, 11). These results strongly suggested that compounds **1** and **2** exhibited significant inhibition effect on the NF-*κ*B pathway activated by LPS.

Taken together, our study demonstrated that the anti-inflammatory mechanism of characteristic oleanane triterpenoids with C-14 carboxyl group (compounds **1** and **2**) from *A. grandis* in LPS-induced RAW 264.7 cells and THP-1 cells for the first time. These two compounds exerted their anti-inflammatory effect by inhibiting the activation of the NF-*κ*B pathway, reducing the phosphorylation of p65 and the nuclear translocation of p65, thereby suppressing the expression of inflammatory-related genes and cytokines. Overall, this work recommends compounds **1** and **2** as potential novel anti-inflammatory agents, and it suggests that these compounds could be therapeutics for inhibiting and treating inflammation-related disorders in the future. Furthermore, additional studies are needed to assess the mechanisms underlying the anti-inflammatory efficacy of compounds **1** and **2** using other cell lines and *in vivo* models.

## Data Availability

The original contributions presented in the study are included in the article/Supplementary Material, further inquiries can be directed to the corresponding authors.
