# Supplementary material for: Oleanane triterpenoids with C-14 carboxyl group from Astilbe grandis inhibited LPS-induced macrophages activation by suppressing the NF-κB signaling pathway
Source: Front Pharmacol. 2024 Aug 1;15:1413876. doi: 10.3389/fphar.2024.1413876 (PMC11324442; doi:10.3389/fphar.2024.1413876)

3α-acetoxyolean-12-en-27-oic acid (**1**):

RAW264.7 cell:

Figure 5A:

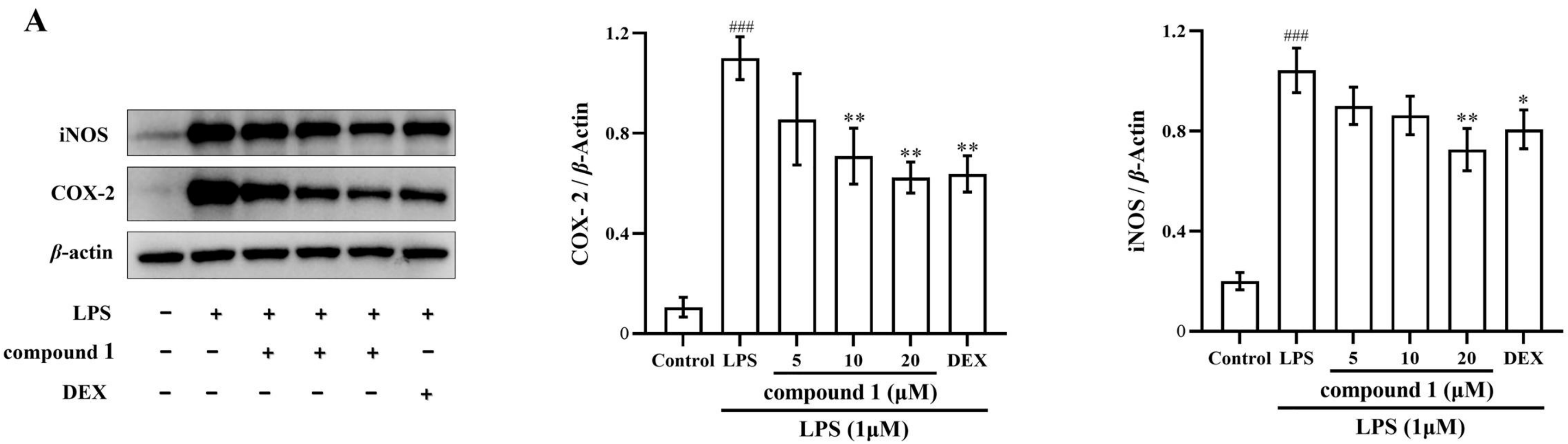

iNOS: 3*α*-acetoxyolean-12-en-27-oic acid (**1**)

Western blot original image

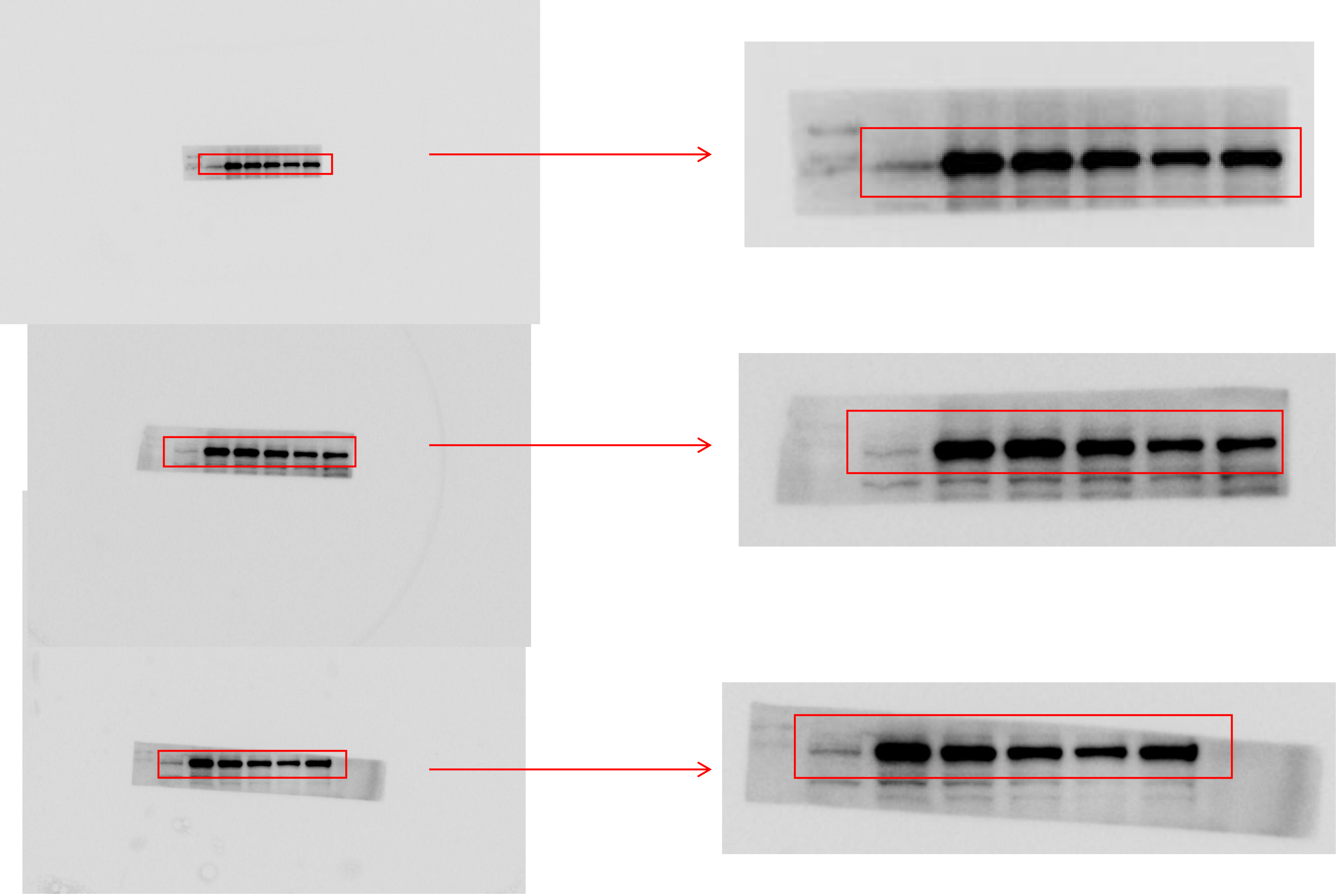

COX-2: 3α-acetoxyolean-12-en-27-oic acid (**1**)

Western blot original image

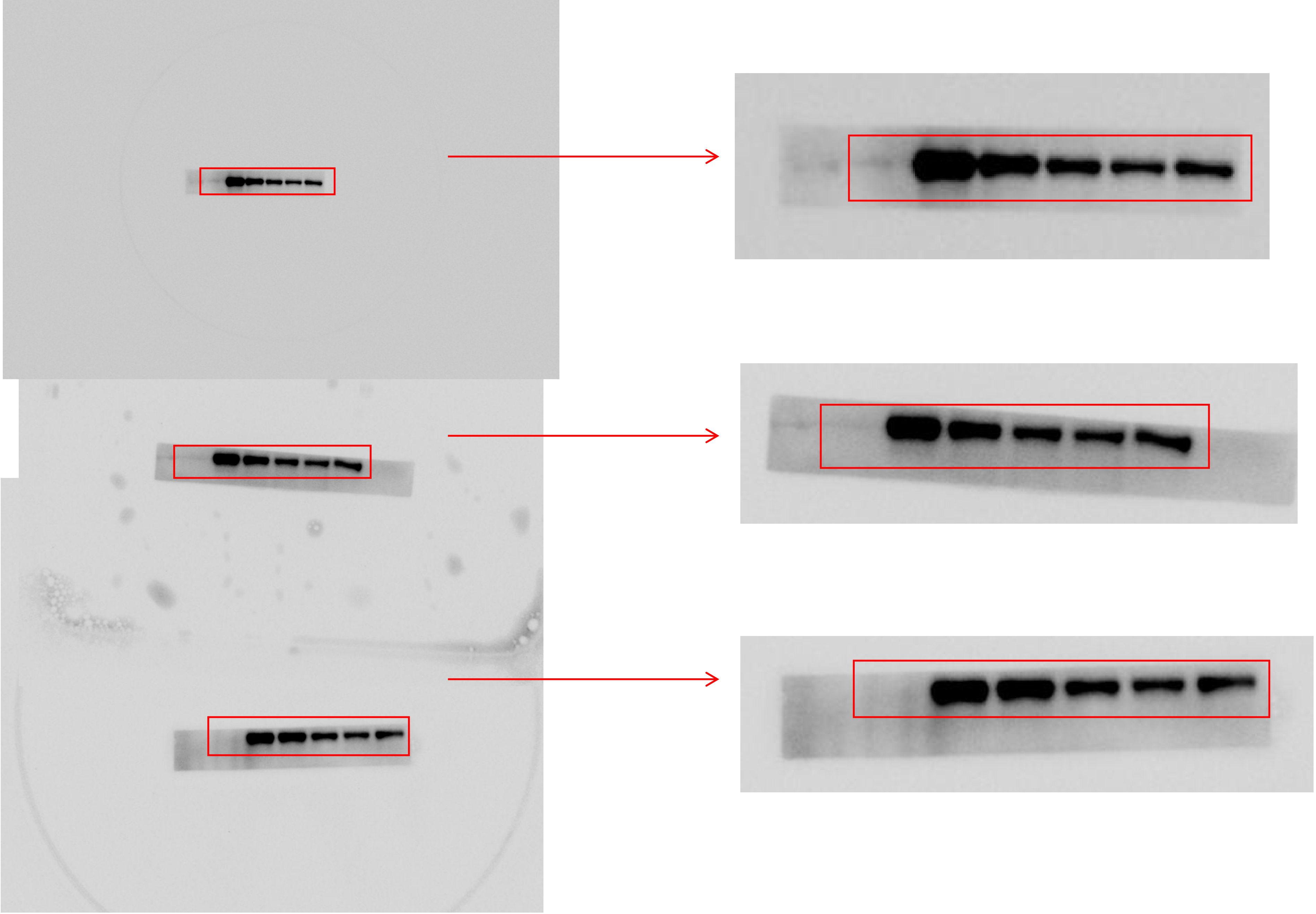

***β*-actin**: 3*α*-acetoxyolean-12-en-27-oic acid (**1**)

Western blot original image

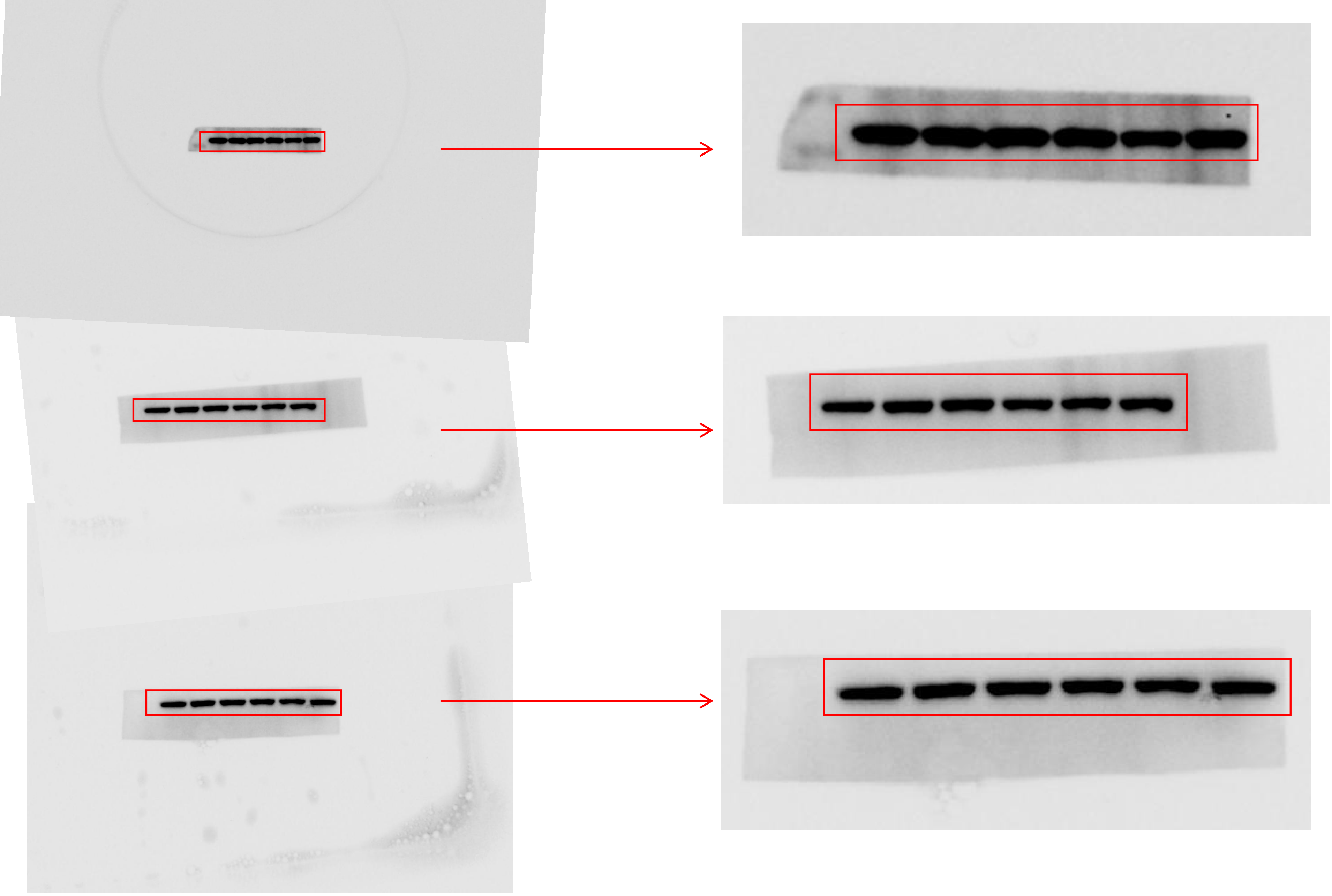

3β-acetoxyolean-12-en-27-oic acid (**2**):

RAW264.7 cell：

Figure 5B:

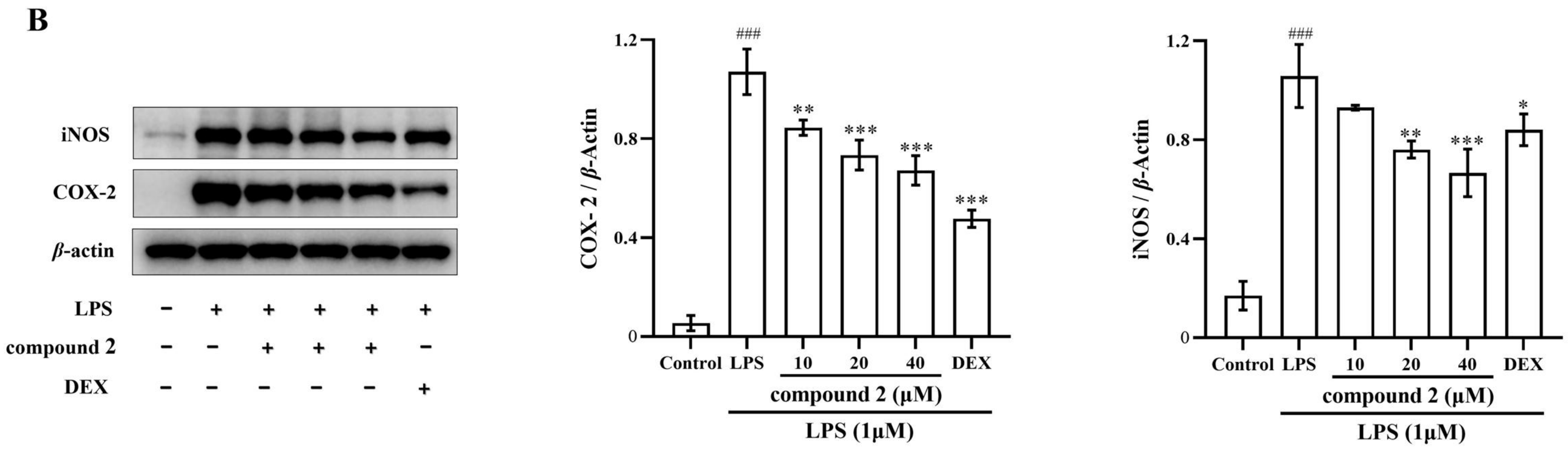

iNOS: 3*β*-acetoxyolean-12-en-27-oic acid (**2**)

Western blot original image

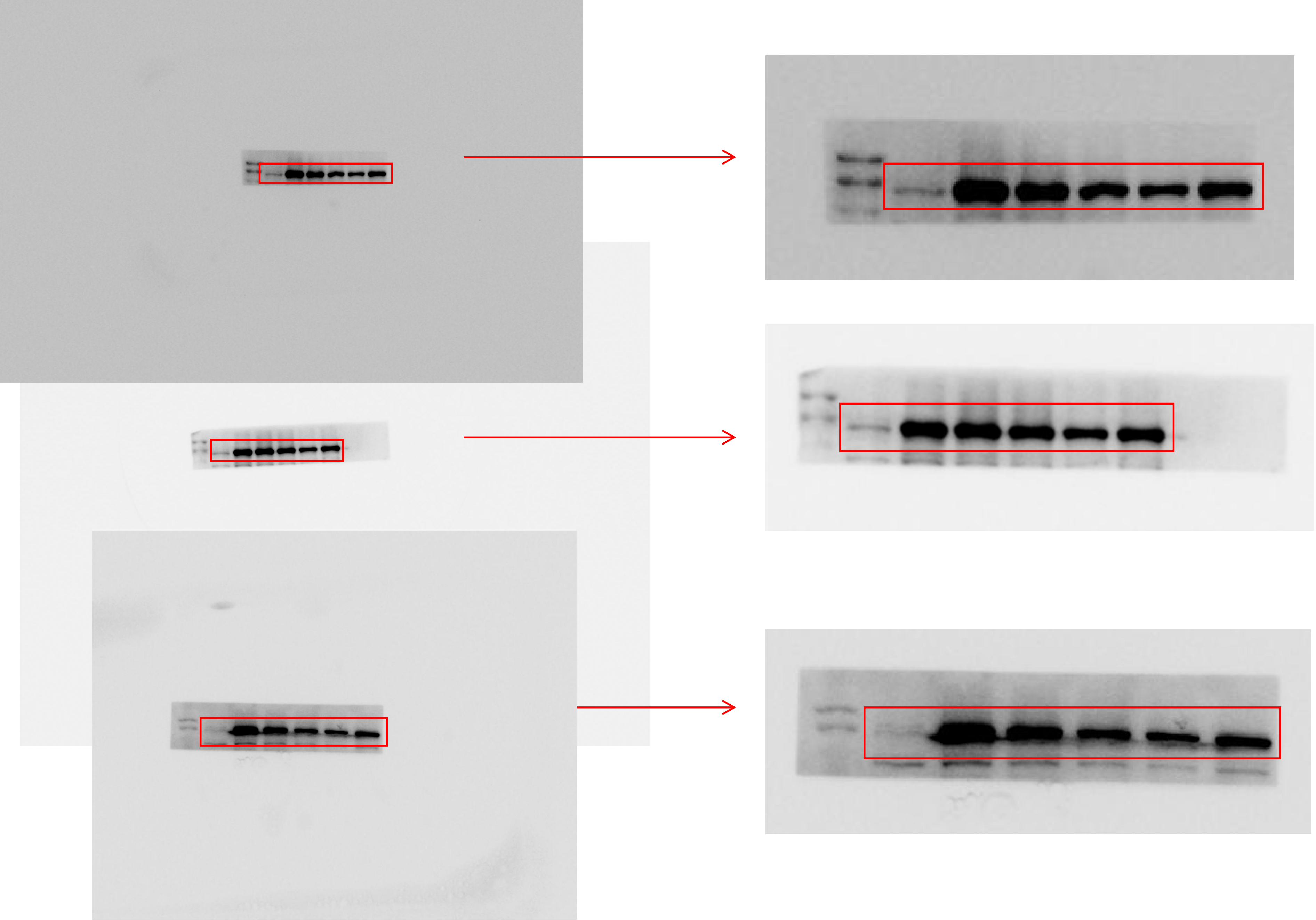

COX-2: 3β-acetoxyolean-12-en-27-oic acid (**2**)

Western blot original image

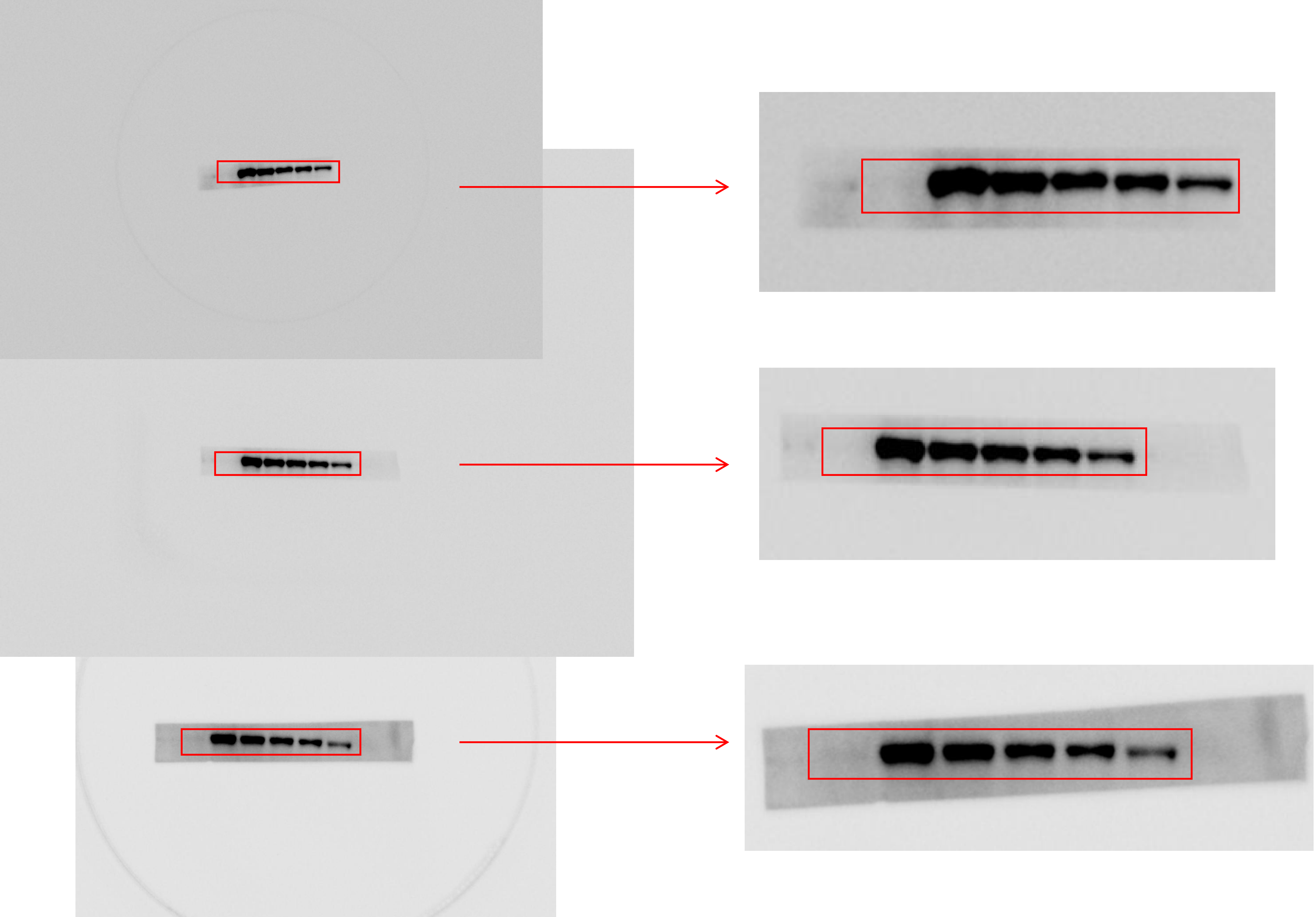

***β*-actin**: 3*β*-acetoxyolean-12-en-27-oic acid (**2**)

Western blot original image

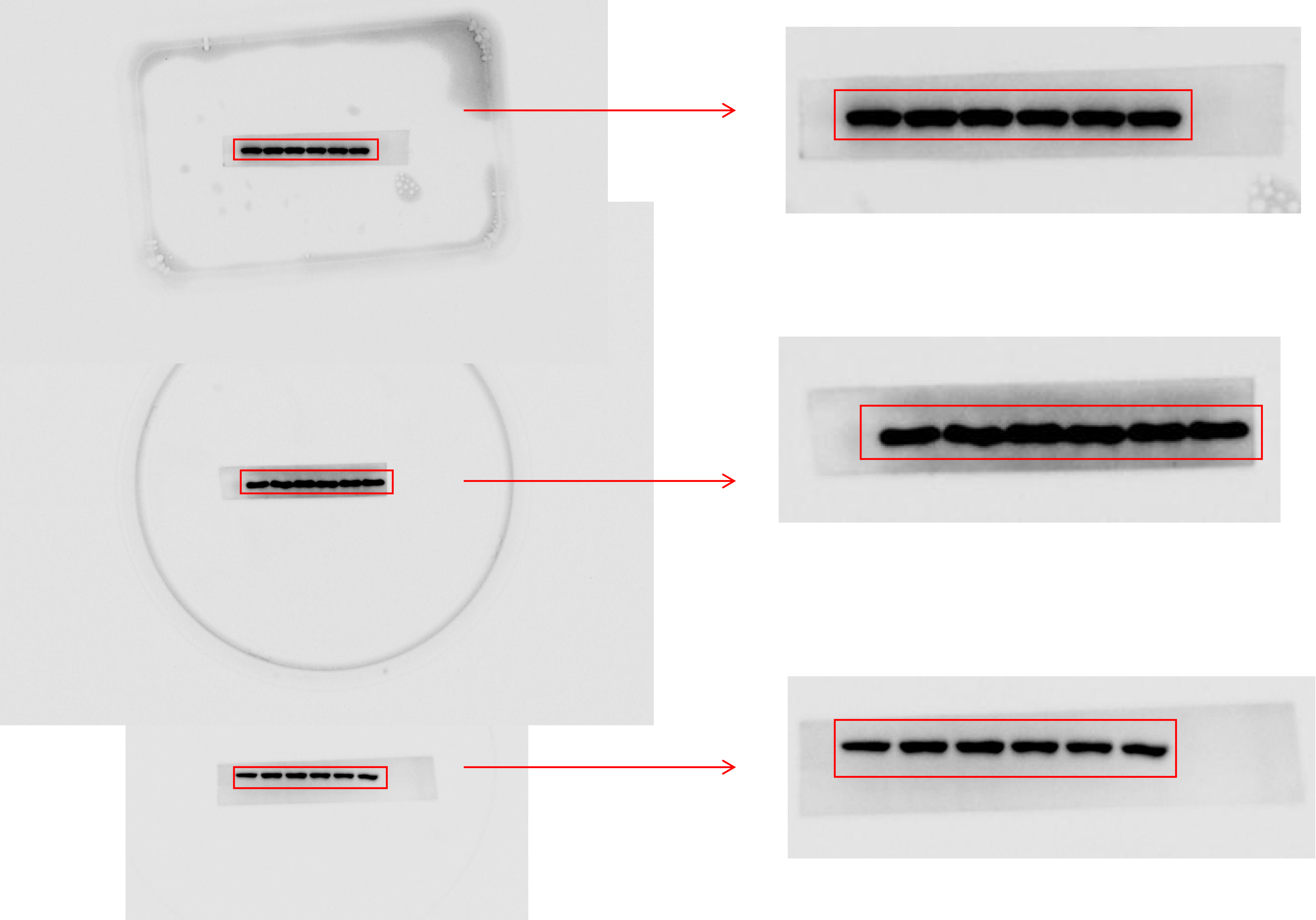

3α-acetoxyolean-12-en-27-oic acid (**1**):

THP-1 cell:

Figure 6A:

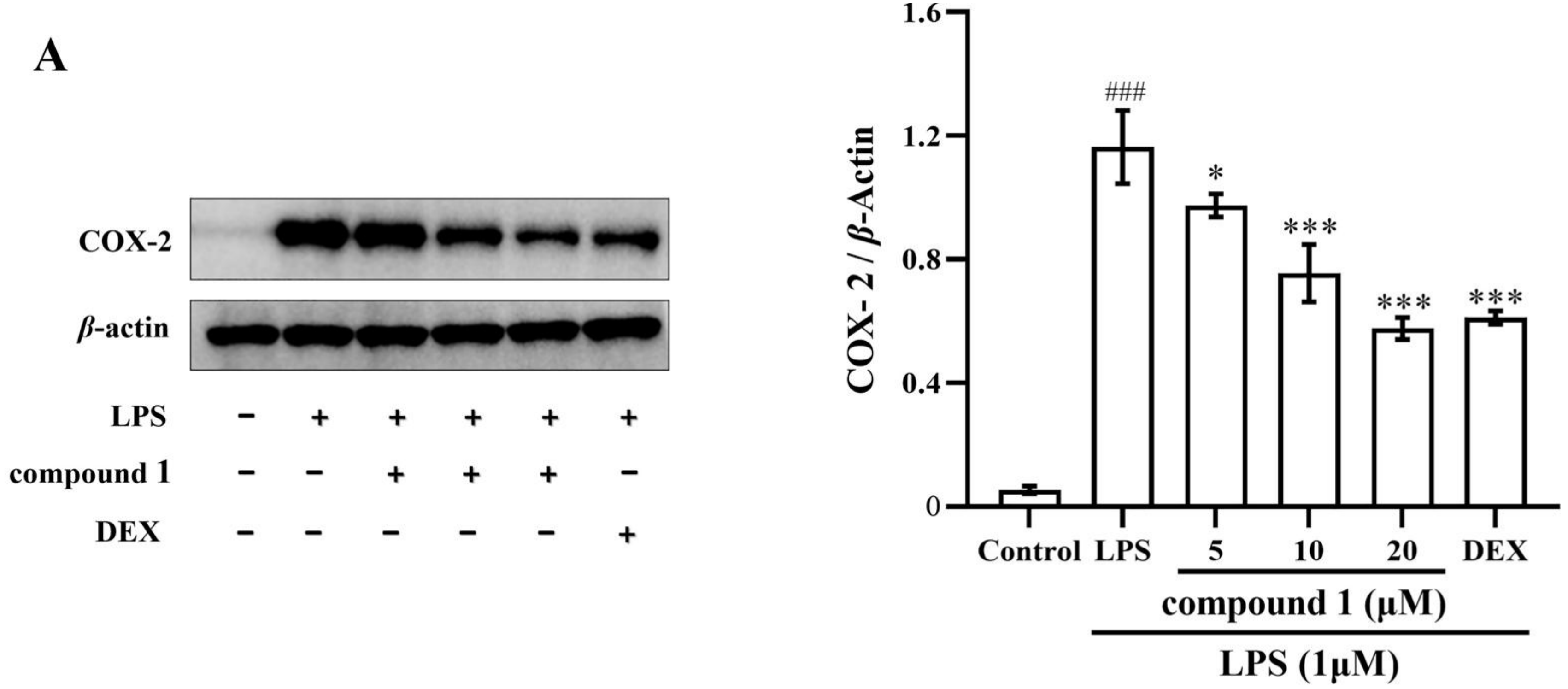

COX-2: 3α-acetoxyolean-12-en-27-oic acid (**1**)

Western blot original image

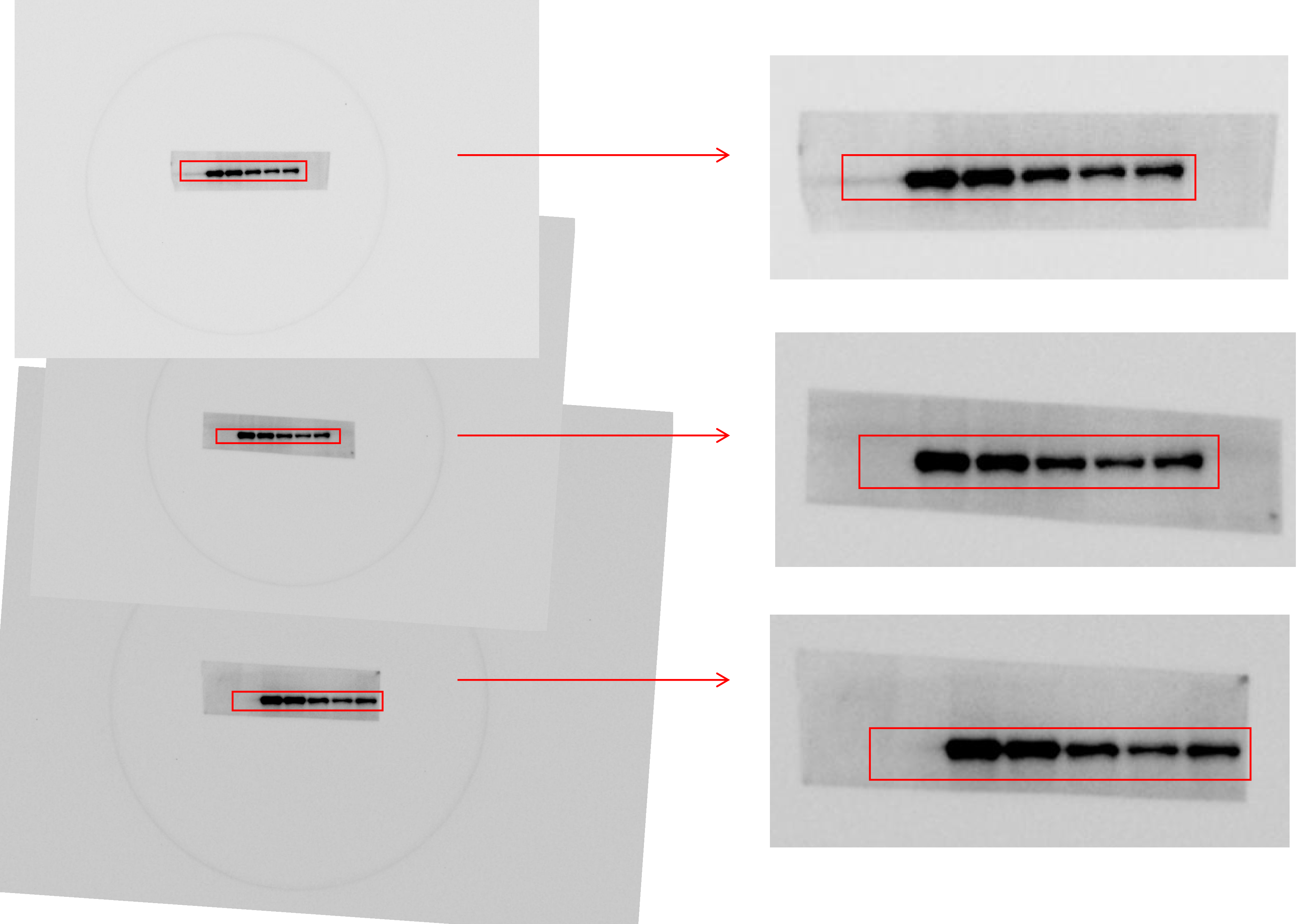

***β*-actin**: 3*α*-acetoxyolean-12-en-27-oic acid (**1**)

Western blot original image

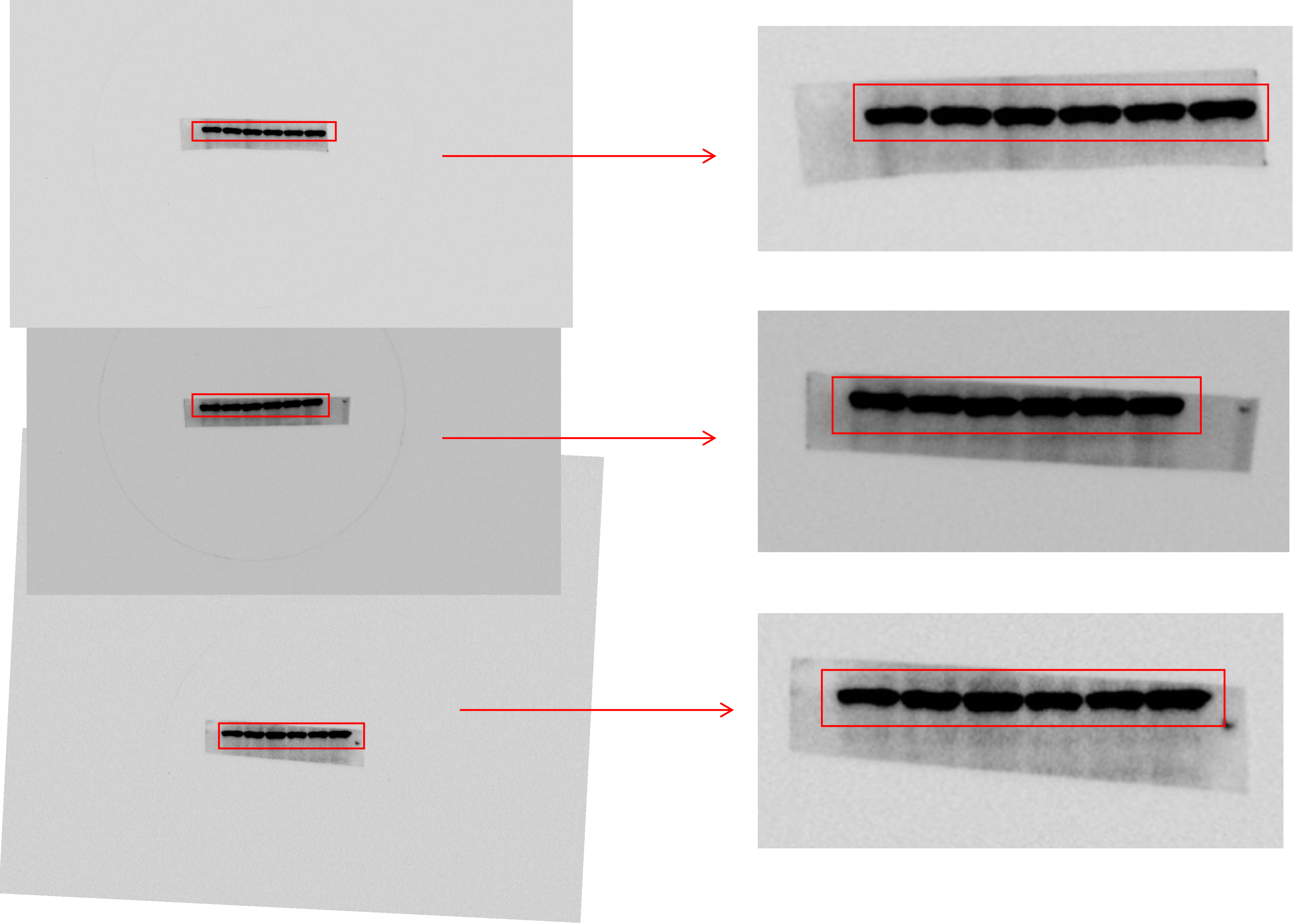

3β-acetoxyolean-12-en-27-oic acid (**2**):

THP-1 cell:

Figure 6B:

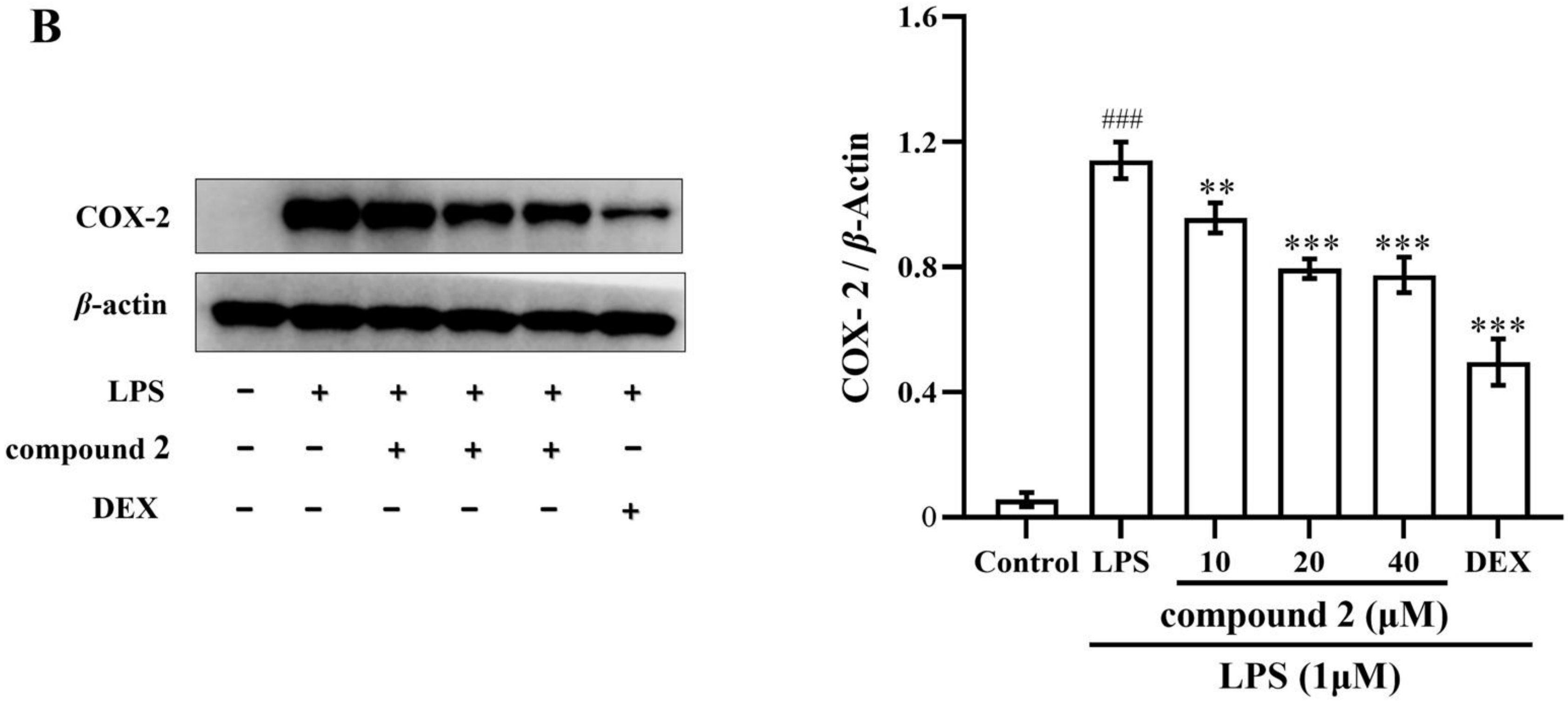

COX-2: 3*β*-acetoxyolean-12-en-27-oic acid (**2**)

Western blot original image

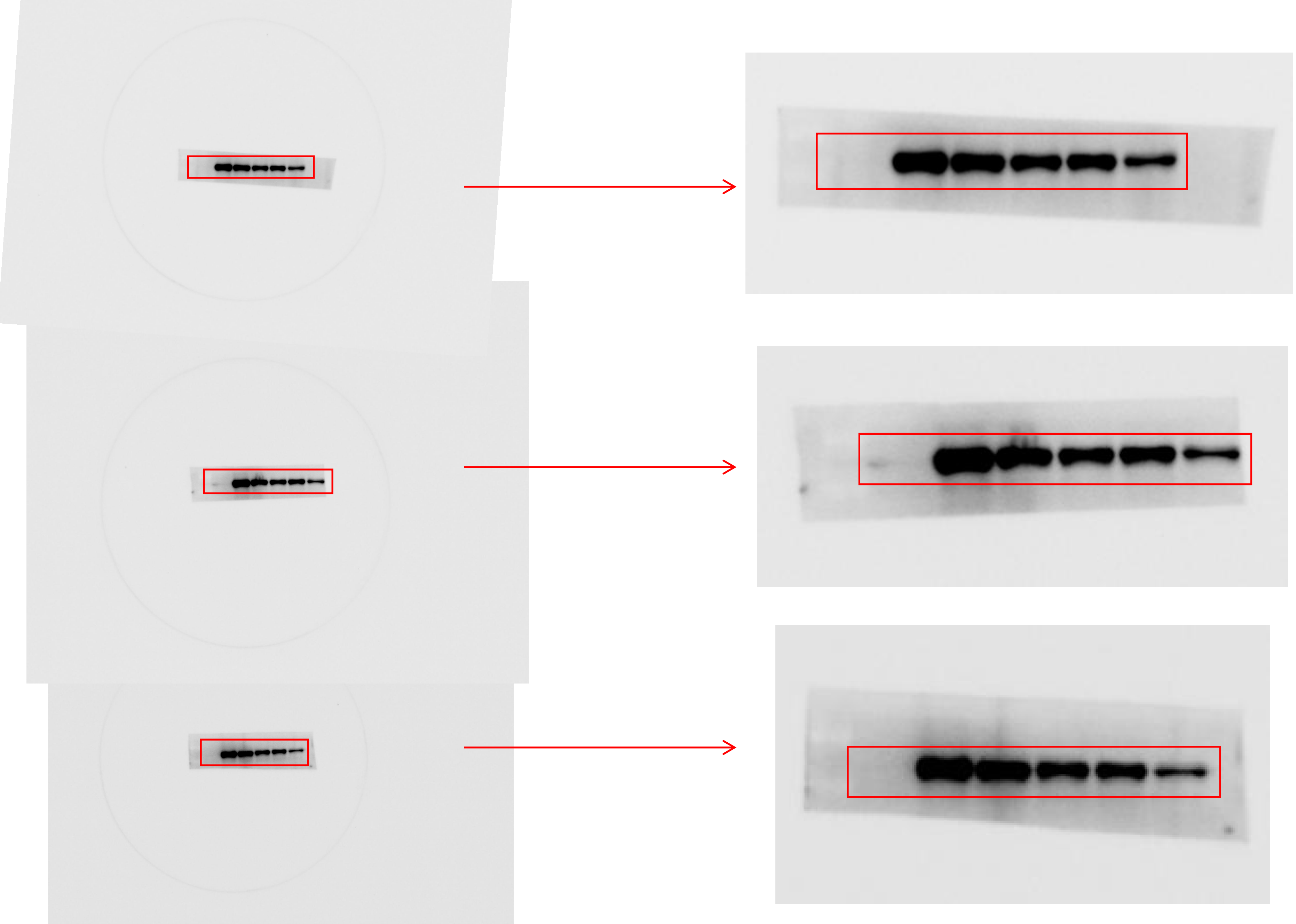

***β*-actin**: 3*β*-acetoxyolean-12-en-27-oic acid (**2**)

Western blot original image

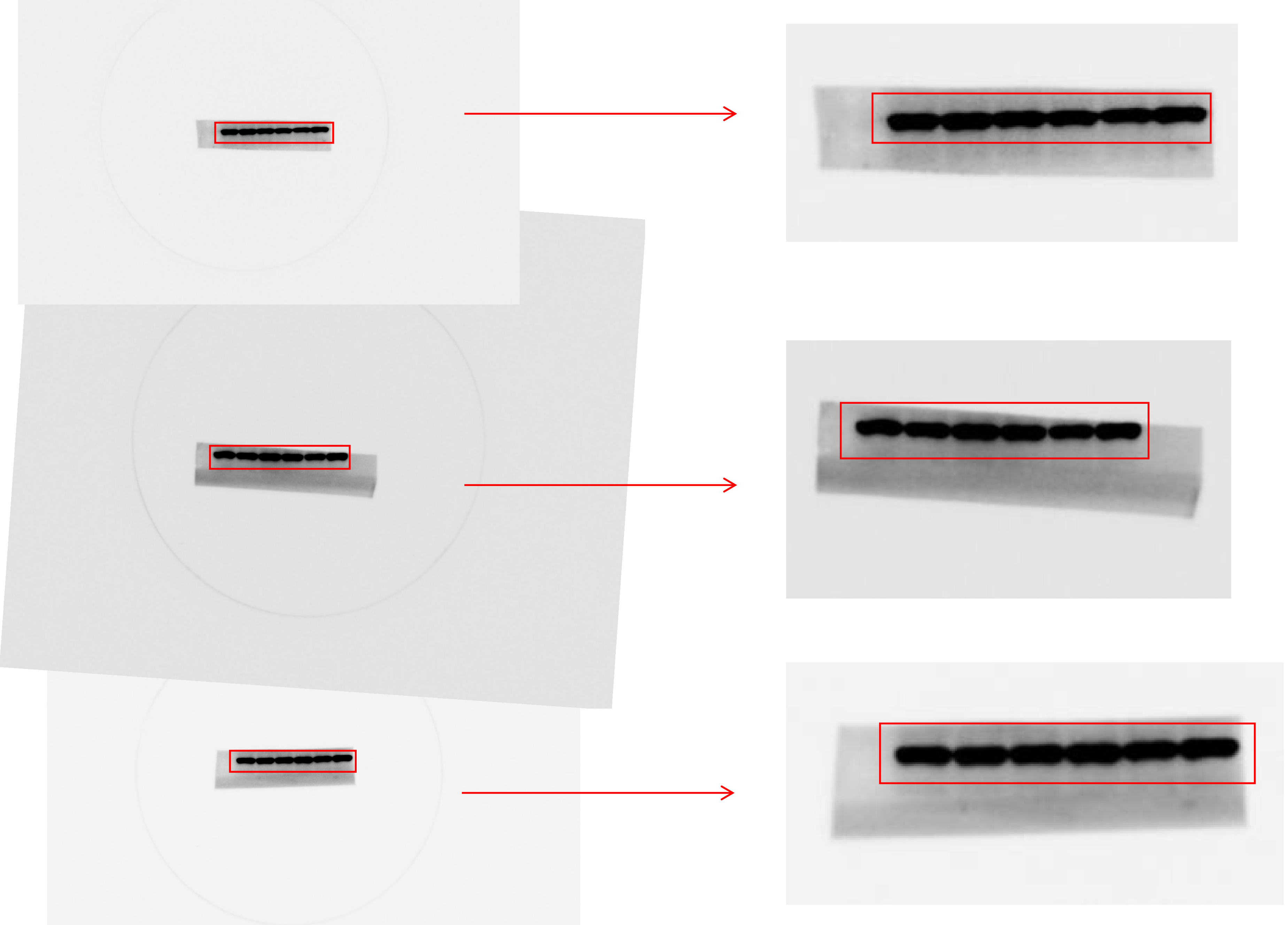

3α-acetoxyolean-12-en-27-oic acid (**1**):

Figure 8A:

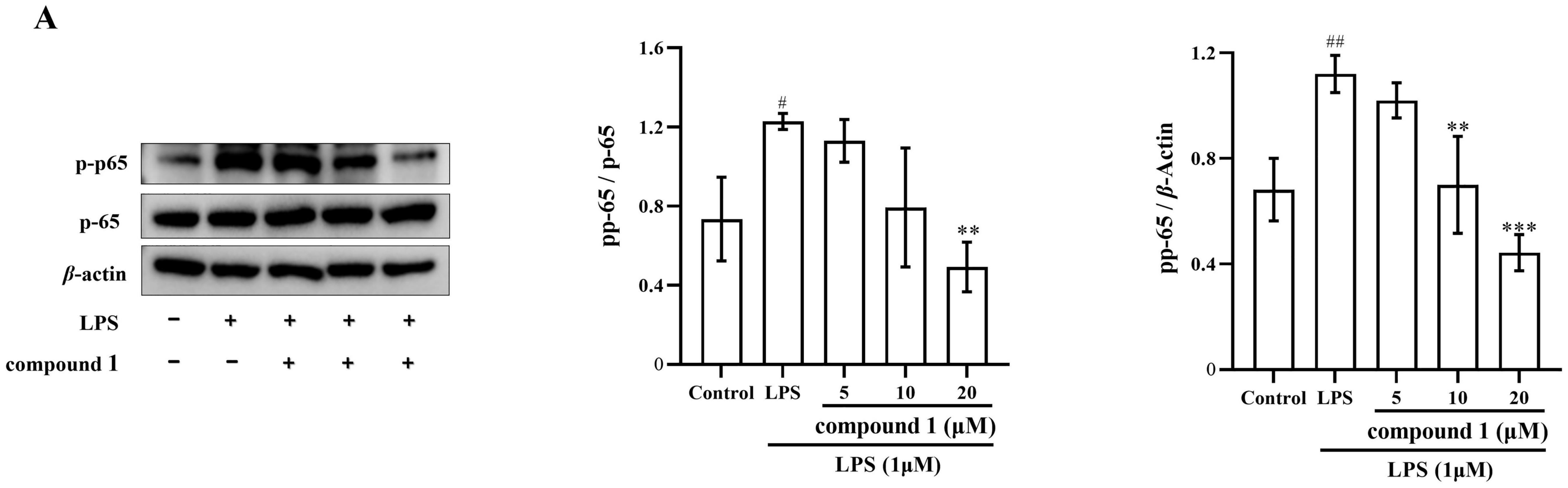

p-p65: 3α-acetoxyolean-12-en-27-oic acid (**1**)

Western blot original image

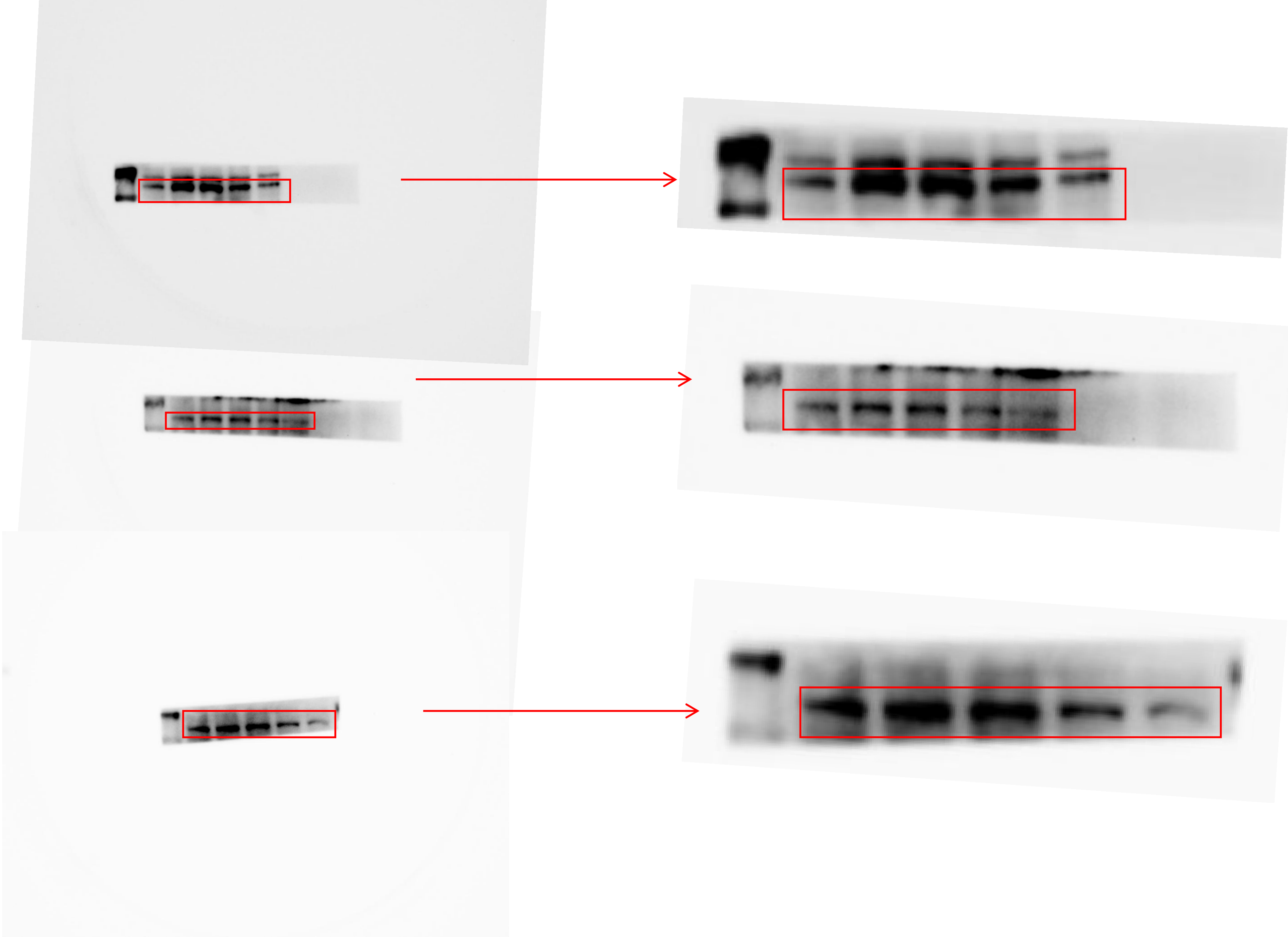

p-65: 3*α*-acetoxyolean-12-en-27-oic acid (**1**)

Western blot original image

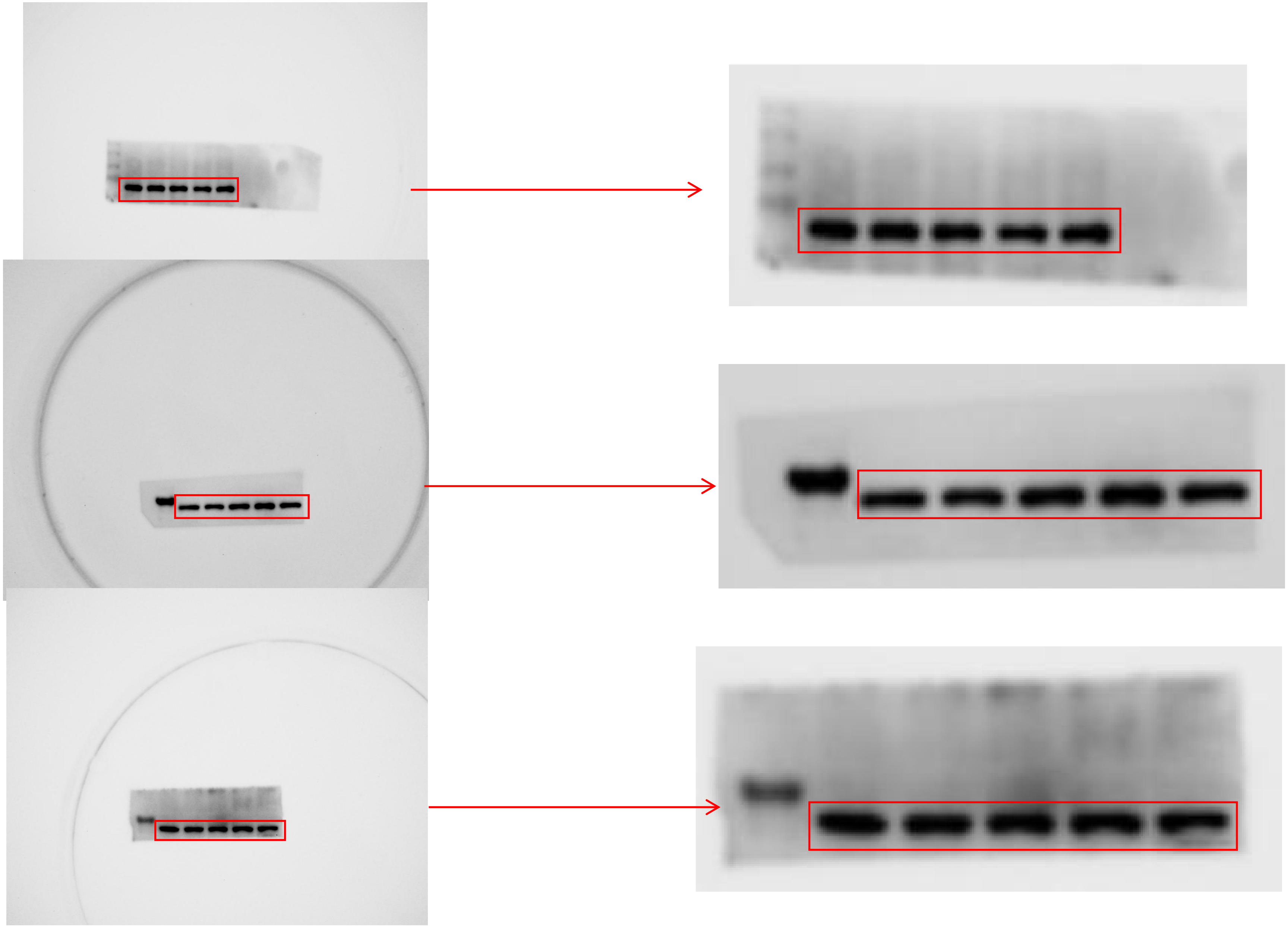

***β*-actin**: 3*α*-acetoxyolean-12-en-27-oic acid (**1**)

Western blot original image

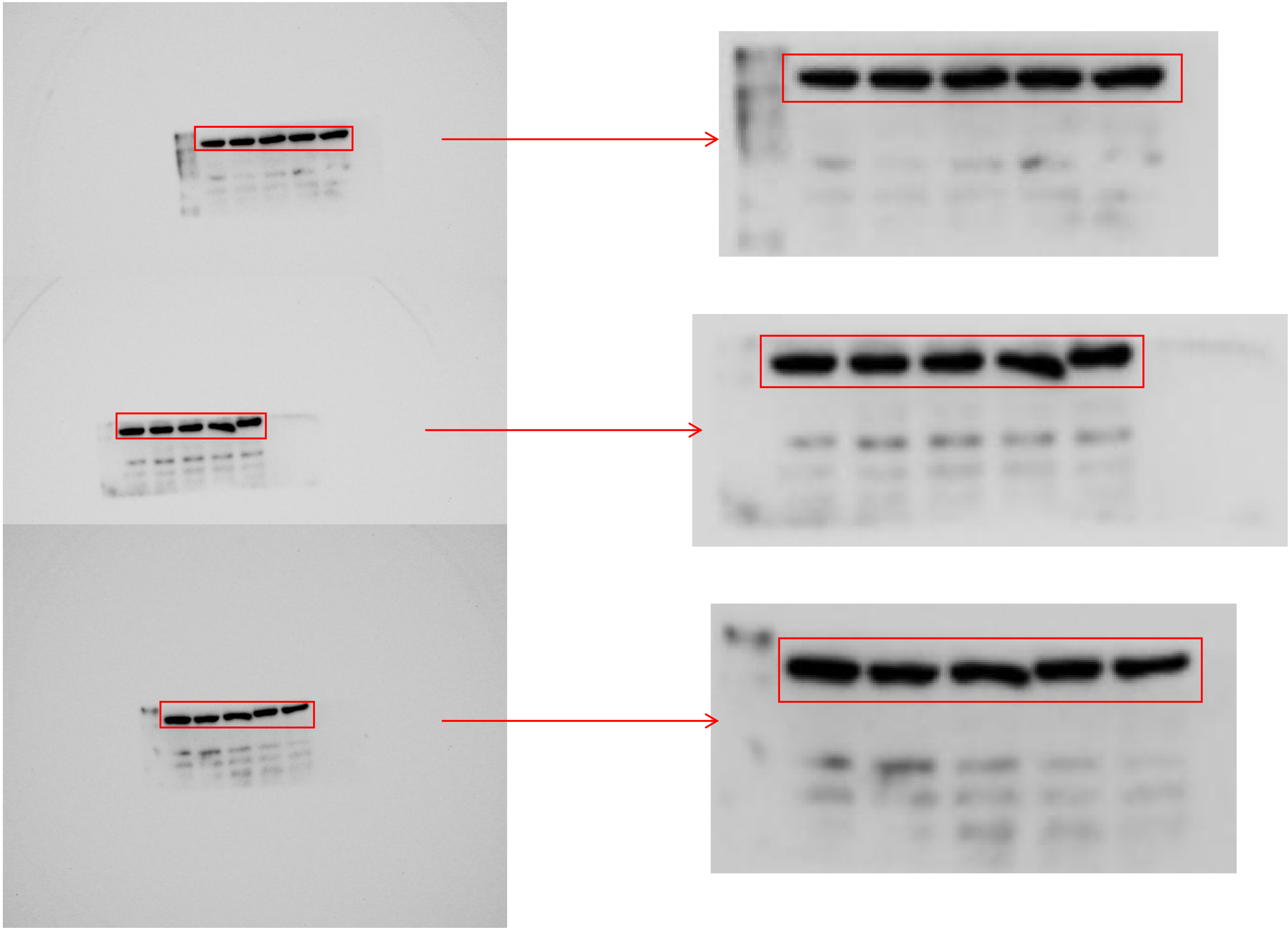

3β-acetoxyolean-12-en-27-oic acid (**2**):

Figure 8B:

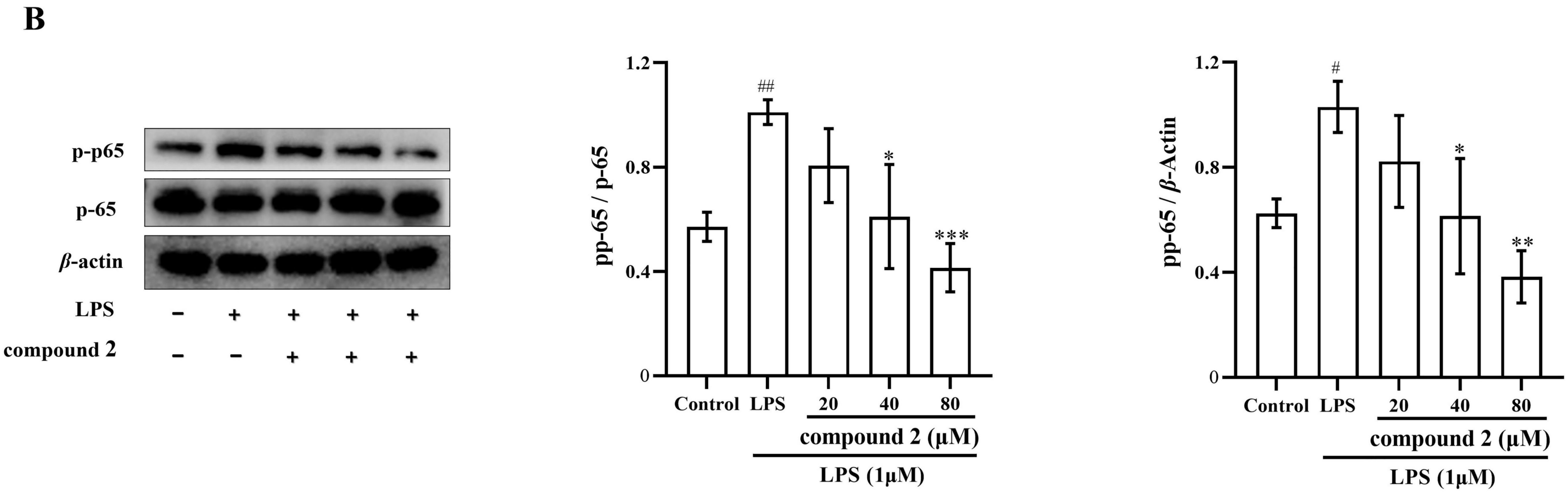

p-p65: 3*β*-acetoxyolean-12-en-27-oic acid (**2**)

Western blot original image

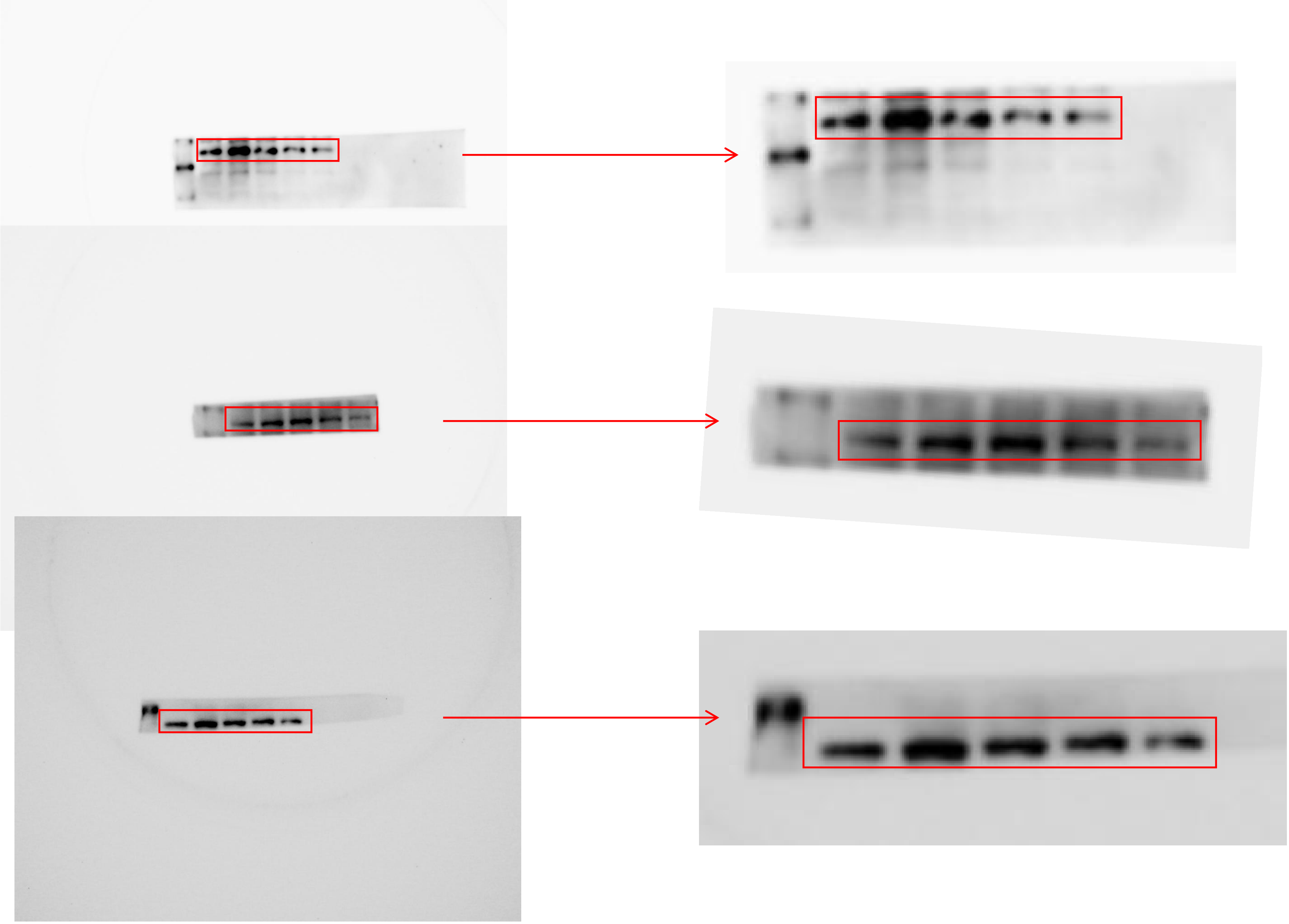

p-65: 3β-acetoxyolean-12-en-27-oic acid (**2**)

Western blot original image

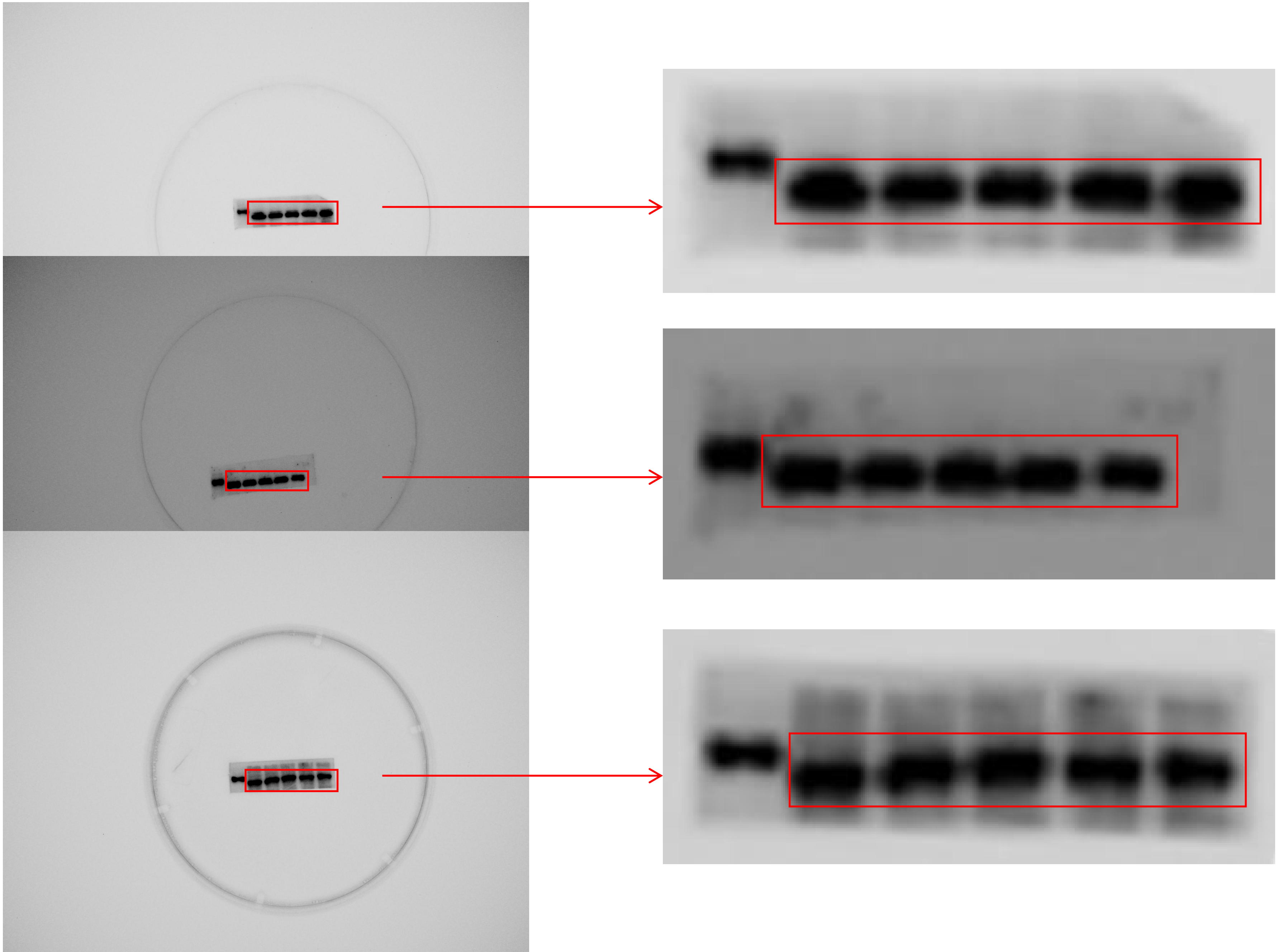

***β*-actin**: 3*β*-acetoxyolean-12-en-27-oic acid (**2**)

Western blot original image

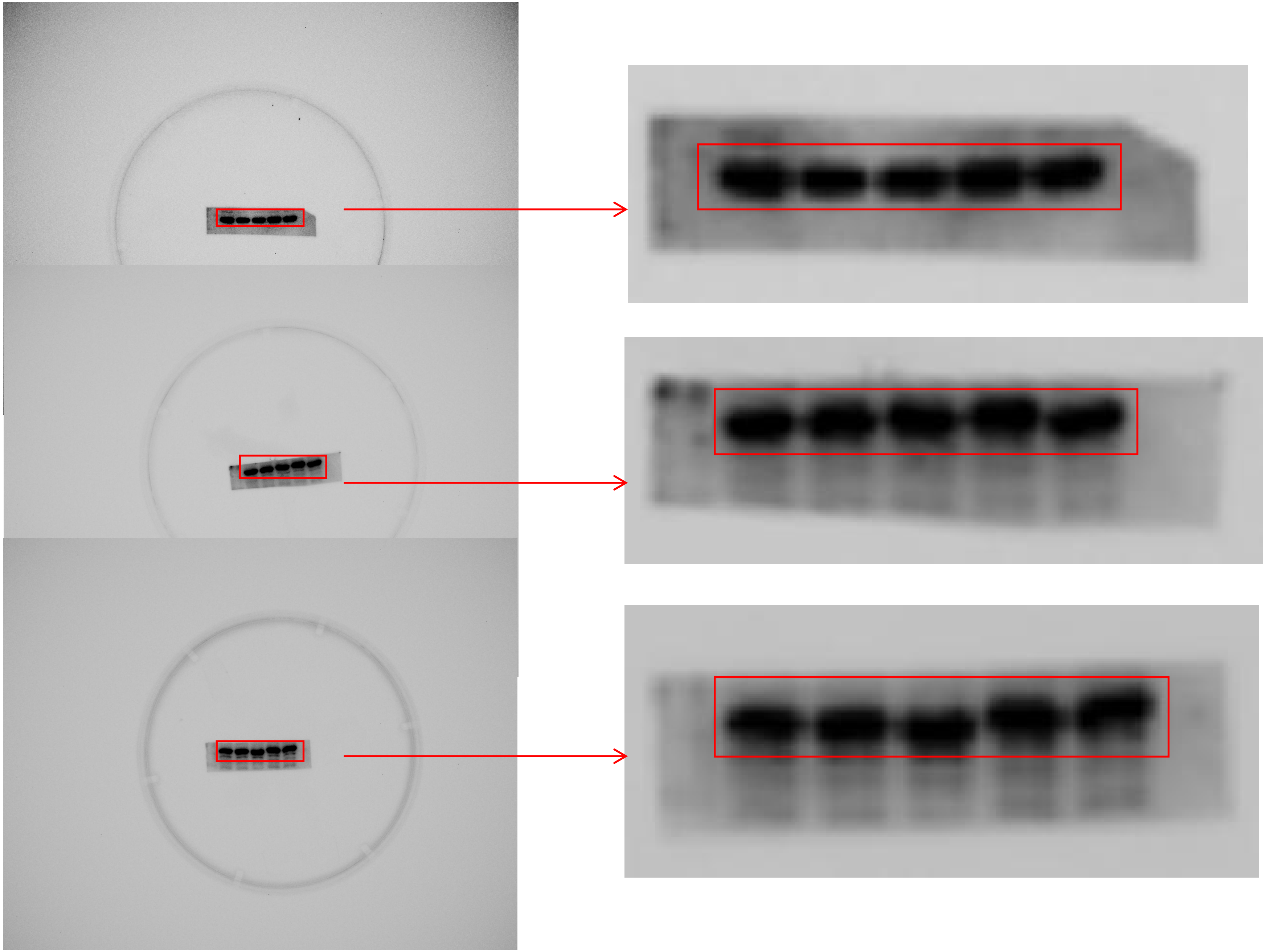

Supplement: Supplementary file 1 [file DataSheet1.PDF]
